# A factor VII-based method for the prediction of anticoagulant response to warfarin

**DOI:** 10.1038/s41598-018-30516-4

**Published:** 2018-08-13

**Authors:** Qing-Xi Ooi, Daniel F. B. Wright, Geoffrey K. Isbister, Stephen B. Duffull

**Affiliations:** 1 School of Pharmacy, University of Otago, Dunedin, New Zealand; 2 School of Medicine and Public Health, University of Newcastle, Newcastle, New South Wales Australia

## Abstract

Warfarin dosing methods based on existing models for warfarin and the international normalised ratio (INR) give biased maintenance dose predictions at the upper and lower quantiles of dose requirements. The aim of this work is to propose a conceptually different approach to predict INR after warfarin dosing. Factor VII concentration was proposed as the principal driving force for the INR. The time to steady-state INR (*t*_*SS*,*INR*_) was determined based on the INR response to changes in factor VII concentrations following warfarin initiation, and from this the steady-state INR (*INR*_*SS*_) was derived. The proposed method requires timed, paired blood samples of INR and factor VII. At different simulated warfarin dose rates, the prediction error associated with the proposed method was shown to be within clinically acceptable limits for both the *t*_*SS*,*INR*_ (±2 days) and *INR*_*SS*_ (±0.2). The use of the method was demonstrated in two patients who were initiated with 5 mg of warfarin daily. The difference in predicted versus actual steady-state INR were 0.0 and −0.4. The proposed method represents a unique approach to predict the INR. It considers factor VII as the main driver for INR and provides valuable information about the time to steady state INR.

## Introduction

Warfarin has been the mainstay of oral anticoagulant therapy for the past several decades. It exerts an anticoagulant effect by inhibiting the recycling of vitamin K hydroquinone in the liver thereby decreasing the production of functional clotting factors II, VII, IX, X, and anticoagulation proteins C, and S. The physiological response to warfarin is a prolonged clotting time. Therapeutically this is useful for the treatment and prevention of thromboembolic disease. The anticoagulant response is monitored clinically using the prothrombin time test which is sensitive to deficiencies in factors II^1^, VII^2^, and X^2^. The ratio of the measured prothrombin time for an individual patient to a value from standardised plasma, adjusted for differences in the tissue factor reagent, is called the International Normalised Ratio (INR).

The safe and effective use of warfarin is dependent on maintaining the INR within a narrow therapeutic range, usually between 2 and 3. The goal of warfarin dose individualisation is therefore to predict the warfarin maintenance dose that will achieve a steady-state INR (*INR*_*SS*_) within this range. Several tools have been developed to aid dose selection in the clinic. While these have been shown to predict warfarin dose requirements on average^3,4^, they are reported to produce biased predictions in patients who require doses in the upper quartile (≥7 mg/day) of warfarin dose requirements^5–8^. In a recently published systematic review and meta-analysis of 22 warfarin dosing tools, the bias in maintenance dose predictions was quantified as an average of −2.3 mg/day^7^. In addition, warfarin dose requirements in patients who require doses in the lower quartile (≤2 mg/day) are also reported to be over-predicted^9,10^.

It appears that current methods do not accurately predict warfarin maintenance doses for patients in the lower or upper quartile of dose requirements. These patients will be those who are at the greatest risk of over- or under-anticoagulation, and who would therefore derive the most benefit from warfarin dose individualisation. The reason for the poor performance of dosing algorithms in the upper and lower quartiles of dose requirements is not understood^7^. It is probable that the empirical models underpinning these dosing methods are too simple to accommodate the complexity of the coagulation system, with many models approximating the relationship between warfarin dose and INR by linear functions^4,11–14^ or an *E*_*max*_ function^15–18^. It is interesting that even the flexibility of a Bayesian forecasting method was insufficient to accurately predict warfarin requirements in those who require higher doses^8^.

A new approach to predict the INR and warfarin dosing requirement is needed. To date, all dosing methods for warfarin rely solely on the measurement of INR, a blunt estimate of anticoagulant response that is a composite of several clotting factors. The INR is easily measured by commercial laboratories and has been directly linked to clinical outcomes of interest^19,20^. This means that it is an attractive biomarker to assess the magnitude of anticoagulation and choice of warfarin dose. However, its interpretation relies heavily on understanding the (complex) mechanistic relationship of INR to warfarin dose. The motivation for this study is the belief that a measure of clotting factor response in addition to INR will better inform the prediction of *INR*_*SS*_, which in turn can be used to accurately predict dosing requirements. In this setting, the clotting factor response provides a signal from the system that lies causally between warfarin exposure and INR. A choice of clotting factor that is particularly sensitive to warfarin would therefore be an appropriate choice to aid interpretation of the observed INR. The manuscript is divided into the following sections: underpinning theory; development of the prediction algorithm; application of the prediction algorithm; and finally, discussion of the proposed algorithm.

## Theory

The proposed approach for the prediction of anticoagulant response to warfarin includes a 2-step process; (1) determining the time to steady-state INR (*t*_*SS*,*INR*_) and (2) from this predicting *INR*_*SS*_ (at *t*_*SS*,*INR*_). The predicted *INR*_*SS*_ could then provide a means of determining maintenance dose requirements.

We define the steady-state INR as the INR that is achieved at the maximum, which is when the system is essentially at equilibrium. It follows that the steady-state INR can be defined mathematically when:

1 $$\begin{array}{c}\,\frac{dINR}{dt}=0;\,t > 0.\,\,\end{array}$$

We note, however, that true steady-state is an asymptotic condition and therefore we relax the definition given in Equation 1 to a practical solution where

2 $$\begin{array}{c}\frac{dINR}{dt}\le {\varepsilon }_{INR};\,t > 0\end{array}$$

and *ε*_*INR*_ is a pre-defined level of tolerance. Here, we define *t*_*SS*,*INR*_ as the smallest value of *t* that satisfies Equation 2.

Once *t*_*SS*.*INR*_ has been determined it is then a matter of deriving *INR*_*SS*_. If, for simplicity, and without loss of generality, we assume warfarin dosing is constant over time, then the solution for *INR*_*SS*_ is a function of *INR*_0_, the INR at baseline, and ${\int }_{0}^{{t}_{SS,INR}}\frac{dINR}{dt}dt$, the cumulative change in INR from baseline to *t*_*SS*,*INR*_.

3 $$\begin{array}{c}IN{R}_{SS}\approx IN{R}_{0}+{\int }_{0}^{{t}_{SS,INR}}\frac{dINR}{dt}\,dt;\,t > 0\,\end{array}$$

Generalising this to variable dosing can be solved by a series of piecewise integrals with break points at times of dose change.

In this work, it is postulated that inclusion of the clotting factor response data in addition to the INR may improve the prediction of *t*_*SS*,*INR*_. INR is a non-linear function of the circulating factors II, VII, and X, and warfarin exposure and is causally linked to the INR via these clotting factors. Of the three clotting factors, factor VII has the shortest degradation half-life, of approximately 6 hours^21–24^, and therefore declines at the greatest rate and is the first to attain a new steady-state concentration. Assuming that:

- simultaneous reduction in factors II, VII, and X leads to less than additive increases in the INR (Assumption 1),
- the most deficient clotting factor drives the INR (Assumption 2),
- under non-steady-state INR conditions, factor VII is always the most deficient (Assumption 3), and
- the non-steady-state INR is the most sensitive to factor VII (Assumption 4),

then, monitoring factor VII in addition to INR will be informative for determination of *t*_*SS*,*INR*_. These assumptions are supported by the widely-accepted notion that factor VII is the principal driving force for the non-steady-state INR^25,26^. In addition, at steady-state INR, there exists a high correlation between the steady-state concentrations of all the vitamin K-dependent factors II, VII and X and hence the steady-state concentration of factor VII would be informative of the eventual steady-state concentrations of the other factors and *INR*_*SS*_. Finally, the assumptions stated above were evaluated in terms of the probability and impact of assumption violation. See Supplementary Information A for details of this analysis.

It is expected that non-steady-state INR is the most sensitive to changes in factor VII. Consequently, the sensitivity of the INR to factor VII should adequately approximate the non-steady-state INR. In this study, the sensitivity of the INR to changes in the concentration of factor VII, denoted here as *SI*_*VII*_, is quantified as the partial derivative of the INR with respect to factor VII:

4 $$\begin{array}{c}S{I}_{VII}({t}_{i})=|\frac{\partial INR({t}_{i})}{\partial VII({t}_{i})}|;\,{t}_{i} > 0.\end{array}$$

Here, *INR* (*n* × 1), *VII* (*n* × 1), and *SI*_*VII*_ (*n* × 1) are vectors over time, *t*_*i*_ (*n* × 1), and $|\,\cdot \,|$ denotes the absolute value. *SI*_*VII*_ values close to zero indicate that INR is insensitive to factor VII whereas larger absolute values depict increased INR sensitivity to factor VII.

Subsequently, the INR, which is approximated by *SI*_*VII*_, can be expressed as a function of *SI*_*VII*_. Similarly, the previously defined steady-state INR status and *t*_*SS*,*INR*_ (Equation 2) as well as *INR*_*SS*_ (Equation 3) can be expressed in terms of *SI*_*VII*_. The steady-state INR status is considered to be achieved when *SI*_*VII*_ reaches its steady-state, accordingly:

5 $$\begin{array}{c}\frac{dS{I}_{VII}}{dt}\le {\varepsilon }_{S{I}_{VII}};\,t > 0\end{array}$$

where ${\varepsilon }_{S{I}_{VII}}$ is analogous to *ε*_*INR*_ although scaled to *SI*_*VII*_. The corresponding expressions for *t*_*SS*,*INR*_ and *INR*_*SS*_ in terms of *SI*_*VII*_ are given below:

6 $$\begin{array}{c}{t}_{SS,INR}\approx \,{\rm{\min }}(t|\frac{dS{I}_{VII}}{dt}\le {\varepsilon }_{S{I}_{VII}});\,t > 0\end{array}$$

7 $$\begin{array}{c}IN{R}_{SS}=IN{R}_{0}+{\int }_{0}^{{t}_{SS,INR}}l(\frac{dS{I}_{VII}}{dt})dt;\,t > 0.\end{array}$$

The steady-state INR, *t*_*SS*,*INR*_, and *INR*_*SS*_, defined from the *SI*_*VII*_ perspective, form the basis of this work.

## Development of the Prediction Algorithm

**Figure 1:**
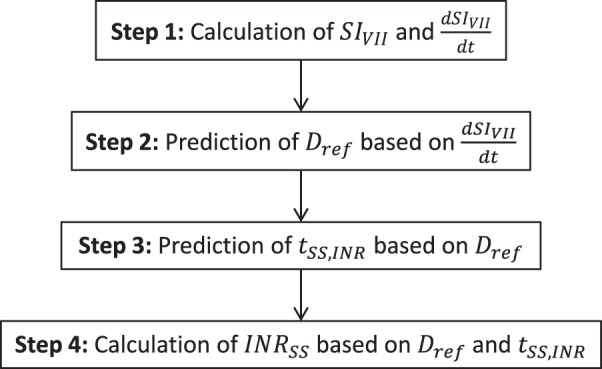
The 4-step workflow to predict *t*_*SS*,*INR*_ and *INR*_*SS*_. *D*_*ref*_ is the typical dose that would yield this value of INR (i.e. sensitivity of the patient to warfarin), *INR*_*SS*_ the steady-state INR, *SI*_*VII*_ the sensitivity index of INR to factor VII, and *t*_*SS*,*INR*_ the time to reach steady-state INR.

In this section, we describe the development of a 4-step algorithm for the prediction of *t*_*SS*,*INR*_ and *INR*_*SS*_. A schematic of the steps is presented in Fig. 1.

In this work, the relationships between warfarin exposure, *SI*_*VII*_, and INR, were derived empirically to match predictions from a quantitative systems pharmacology (QSP) model of the coagulation network^27^. This is a heuristic model-order reduction technique in which an empirical approximation to predictions from a QSP model of the coagulation network is developed. Using this approach, the mechanistic behaviour of the system over the range of simulations from the QSP were captured, albeit the mechanistic nature of the relationships is not retained. It is worth noting that, implicit to this approach, it is assumed that:

- the QSP coagulation network model^27^ is adequate in describing the warfarin-clotting factors-INR relationship (Assumption 5) and that
- the simulated clotting factors-INR time course is representative of that of typical patients initiated with warfarin (Assumption 6).

Of primary interest to the evaluation of these assumptions, is the predictive performance of the QSP coagulation network model. The model was previously evaluated and shown to perform well in characterising the INR based on the clotting factor profiles of 20 patients^27,28^. Additionally, in this work, predictions from the QSP coagulation network model were compared to available time course data of factors II, VII, X, and INR for 17 warfarin patients^29,30^. The model predictions were in reasonable agreement with the observed data thereby suggesting that the QSP coagulation network model is likely to be adequate in characterising the warfarin-clotting factors-INR relationship (see Supplementary Figure S3). More details of the evaluation of these assumptions can be found in Supplementary Information A.

### Calculation of ***SI***_***VII***_ and $\frac{{\boldsymbol{dS}}{{\boldsymbol{I}}}_{{\boldsymbol{VII}}}}{{\boldsymbol{dt}}}$

**Figure 2:**
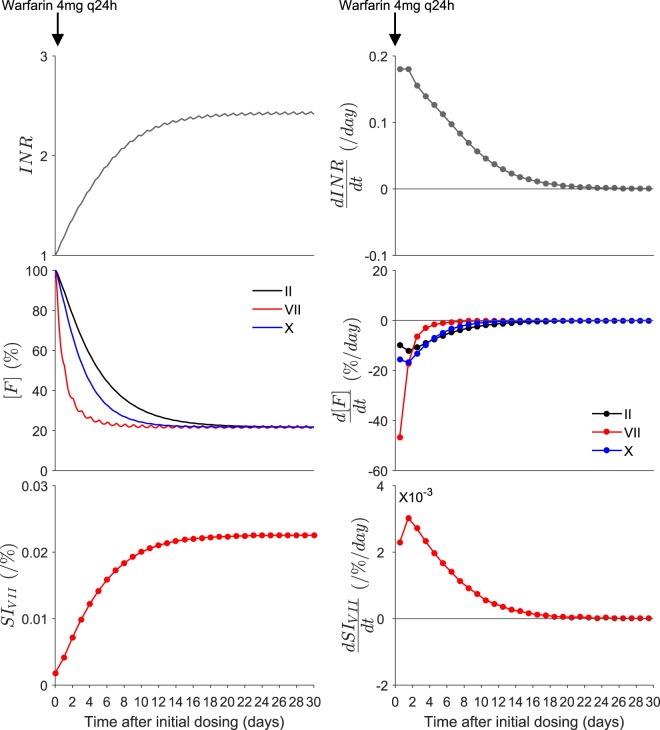
The left hand panels show the time course of INR, factors II, VII, and X, and *SI*_*VII*_ following initiation of 4 mg warfarin daily predicted from the QSP model. The right-hand panels show the corresponding derivative plots with respect to time for INR, and factors II, VII and X, and *SI*_*VII*_. *SI*_*VII*_ refers to the sensitivity index of INR to factor VII. Note the units of the factor concentrations are % of change from baseline.

The time course of factors II, VII, and X, and INR for a typical patient commenced on warfarin doses of 1 mg, 4 mg, 7 mg, 10 mg, and, 13 mg daily were simulated from a previously developed mathematical model of the coagulation network in MATLAB (v.R2015a, The MathWorks Inc., Natick, Massachusetts, USA)^27^. *SI*_*VII*_ was quantified according to (Equation 4) over *t*_*i*_(*n* × 1). A representative time course of factors II, VII, X, INR, *SI*_*VII*_ and their corresponding derivatives for a typical patient commenced on warfarin 4 mg daily is shown in Fig. 2. It is observed that the time course of change in *SI*_*VII*_ is similar to that of the INR.

**Figure 3:**
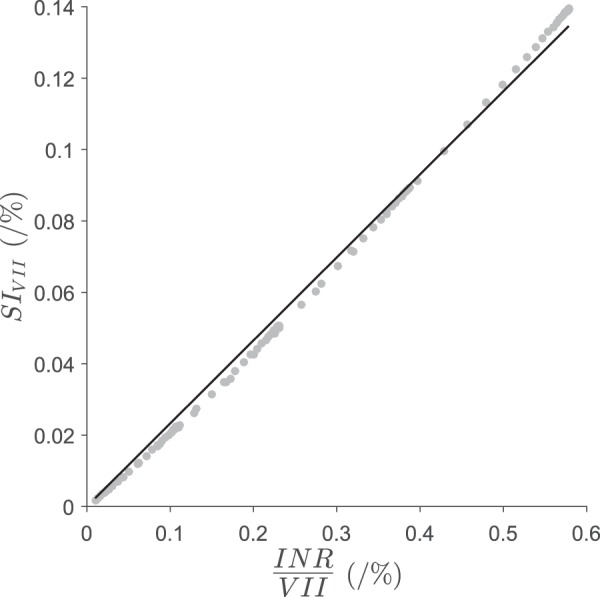
Approximating *SI*_*VII*_ using the ratio of INR-to-factor VII. The solid line is the model prediction from a one-parameter linear model (Equation 8) with slope, *q* = 0.233 and the filled circles are the data vectors for *SI*_*VII*_ and INR-to-factor VII ratio that were simulated from the coagulation network model. *SI*_*VII*_ is the sensitivity index of INR to factor VII. The units of factor VII here are % of change from baseline and INR is dimensionless.

For clinical application, a blood sample of both factor VII and INR is needed for the calculation of *SI*_*VII*_ and $\frac{dS{I}_{VII}}{dt}$. To allow determination of the derivative from a single sample, a steady state approximation was developed (note that factor VII achieves steady state rapidly compared to other factors),

8 $$\begin{array}{c}S{I}_{VI{I}_{{t}_{i}}}\approx q\times \frac{IN{R}_{{t}_{i}}}{VI{I}_{{t}_{i}}};\,{t}_{i}\ge {t}_{SS,VII}.\end{array}$$

here, *q* is the proportionality constant and *t*_*i*_ is the time after warfarin dose change (with index *i* to denote a particular sampling time). A review of the relationship shown in Equation 8 is shown in Fig. 3. The data were fitted in MATLAB using the “fitnlm” algorithm. The proportionality constant, *q*, was estimated to be 0.233 with a relative standard error (RSE) of 0.3%. The INR-to-factor VII ratio has intuitive appeal whereby a high INR-to-factor VII ratio indicates a high sensitivity of INR to factor VII, and vice versa, which is consistent with the original interpretation of *SI*_*VII*_. Finally, using the INR-to-factor VII ratio as an approximation to *SI*_*VII*_, the $\frac{dS{I}_{VII}}{dt}$ can now be derived from paired, timed samples of factor VII and INR. Higher order models were not considered given the need for more blood samples and the acceptable assumption of linearity.

9 $$\begin{array}{c}\frac{dS{I}_{VII}}{dt}\approx \frac{S{I}_{VI{I}_{{t}_{2}}}-S{I}_{VI{I}_{{t}_{1}}}}{{t}_{2}-{t}_{1}};\,{t}_{2} > {t}_{1}\ge {t}_{SS,VII}.\end{array}$$

### Prediction of *D*_*ref*_

From $\frac{dS{I}_{VII}}{dt}$, an individualised measure of the typical dose, *D*_*ref*_, can be obtained. *D*_*ref*_ quantifies the sensitivity of the patient to warfarin in terms of the dose that would be taken by a typical patient to achieve the same level of anticoagulant response. For instance, if a patient is particularly sensitive to warfarin therapy then a standard warfarin dose (e.g. *D* = 4 *mg*/*day*) will lead to an exaggerated anticoagulant response, which normally takes a much higher dose to achieve in a typical patient (e.g. *D*_*ref*_ = 10 *mg*/*day*). In other words, if a patient is sensitive to warfarin then ${D}_{ref} > D$, if the patient displays similar sensitivity to that of a typical patient then ${D}_{ref}\approx D$, and if the patient is less sensitive then ${D}_{ref} < D$. The deviation of *D*_*ref*_ from *D* therefore quantifies the difference of the patient from the typical patient. See Supplementary Information B for a summary.

**Figure 4:**
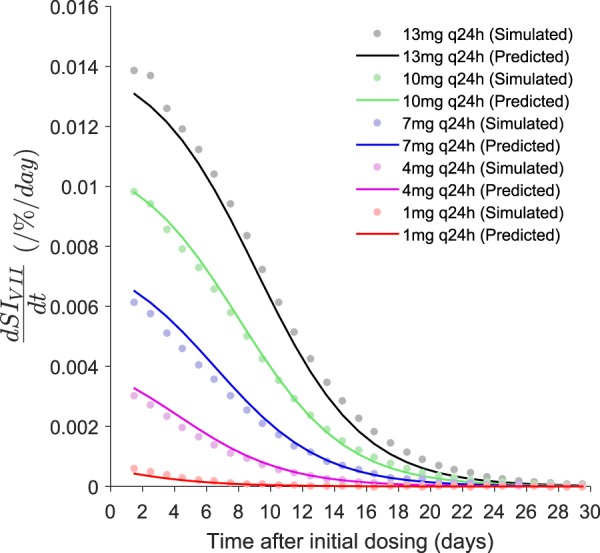
Model fits of the dose-dependent general logistic function for the time course of $\frac{dS{I}_{VII}}{dt}$. The solid lines are the predictions from the empirical logistic function (Equation 11) and the filled circles are the data vectors for $\frac{dS{I}_{VII}}{dt}$ that were simulated from the QSP coagulation network model. Data beyond the model predictions are omitted from the figure. *SI*_*VII*_ is the sensitivity index of INR to factor VII.

The value of *D*_*ref*_ relates to the dose-dependency of *SI*_*VII*_ and $\frac{dS{I}_{VII}}{dt}$ and no closed form function is available for this relationship from the mechanistic model. A function was, therefore, derived empirically. A model for the time course of $\frac{dS{I}_{VII}}{dt}$ was developed based on simulated time courses of $\frac{dS{I}_{VII}}{dt}$ at different values of *D*_*ref*_ (at doses of 1 mg, 4 mg, 7 mg, 10 mg, and, 13 mg daily). In each instance, this was for a typical patient. The time course of $\frac{dS{I}_{VII}}{dt}$ values from the QSP model were fitted using a 3-parameter logistic function such that:

10 $$\begin{array}{c}\frac{dS{I}_{VII}}{dt}=\frac{h}{1+{e}^{p\times (t-g)}};\,t > 0.\end{array}$$

here, *h* is the upper horizontal asymptote, *p* is the shape parameter, and *g* is the magnitude of horizontal shift. Both *h* and *g* are functions of *D*_*ref*_ and *p* is considered independent. The full model expression is given in Equation 11 and the final parameter estimates are given in Table 1 (see Supplementary Information C for details of the individual models developed for data simulated at different warfarin dosing rates).

11 $$\begin{array}{rcl}\frac{dS{I}_{VII}}{dt} & = & \frac{h({D}_{ref})}{1+{e}^{p\times (t-g({D}_{ref}))}};\,t > 0\\ h({D}_{ref}) & = & {a}_{0}+{a}_{1}\times {D}_{ref}\\ g({D}_{ref}) & = & {b}_{0}+{b}_{1}\times \,\mathrm{ln}\,{D}_{ref},\end{array}$$

where, *a*_*i*_ and *b*_*i*_ are parameters that define the dose-dependency of *h* and *g*. See Fig. 4 for model fit. A symbolic expression for *D*_*ref*_ from Equation 11 was solved numerically.

**Table 1:** Parameter estimates of the logistic function for the time course of $\frac{dS{I}_{VII}}{dt}$.

Function	Final estimate (%RSE)		*r* ^2^
$\frac{dS{I}_{VII}}{dt}$	*p*		—
	0.300 fixed		
*h*(*D*_*ref*_)	*a*_0_ (/%/day)	*a*_1_ (/%/mg)	0.988
	0.000392 (123)	0.00108 (5.46)	
*g*(*D*_*ref*_)	*b*_0_ (days)	*b*_1_ (days)	0.995
	−1.48 (17.9)	4.13 (3.42)	

Both *h* and *g* are dose-dependent and *p* is considered independent. At different warfarin dosing rates, the estimates for *p* obtained were largely similar i.e. ranging from 0.260 to 0.301 and fixing of *p* to 0.300 resulted in an almost identical model fit. *a*_*i*_ defines the dose-dependency of *h*, *b*_*i*_ defines the dose-dependency of *g*, *D*_*ref*_ is the warfarin daily dose for a typical patient, *h* the upper horizontal asymptote, *g* the magnitude of horizontal shift, *p* the shape parameter, *SI*_*VII*_ the sensitivity index of INR to factor VII, *r*^2^ the adjusted coefficient of determination, and *RSE* the relative standard error.

### Calculation of *t*_*SS,INR*_

An expression for *t*_*SS*,*INR*_ that is now dependent on *D*_*ref*_ is obtained by combining Equations 6 and 11:

12 $$\begin{array}{rcl}{t}_{SS,INR} & \approx  & {\rm{\min }}(t|\frac{h({D}_{ref})}{1+{e}^{p\times (t-g({D}_{ref}))}}\le {\varepsilon }_{S{I}_{VII}});\,t > 0\,\\ h({D}_{ref}) & = & {a}_{0}+{a}_{1}\times {D}_{ref}\\ g({D}_{ref}) & = & {b}_{0}+{b}_{1}\times \,\mathrm{ln}\,{D}_{ref}.\end{array}$$

From Equation 5, a pre-requisite to the calculation of *t*_*SS*,*INR*_ is the choice of a suitable value of ${\varepsilon }_{S{I}_{VII}}$. The choice of ${\varepsilon }_{S{I}_{VII}}$ should correctly classify a given INR as non-steady-state (or steady-state) when the INR is truly non-steady-state (or steady-state). The choice of ${\varepsilon }_{S{I}_{VII}}$ was based on optimising the receiver operating characteristic (ROC). Details of the method are provided in Supplementary Information D. It is seen that a choice of ${\varepsilon }_{S{I}_{VII}}=0.000150$ provides the optimal operating characteristics of the error tolerance.

### Calculation of *INR*_*SS*_

*INR*_*SS*_ can be determined based on the calculated *t*_*SS*,*INR*_ if the function $l\,(\frac{dS{I}_{VII}}{dt})$ in Equation 7 has an explicit expression. Again, no mechanistic function is available and a function was derived empirically.

**Figure 5:**
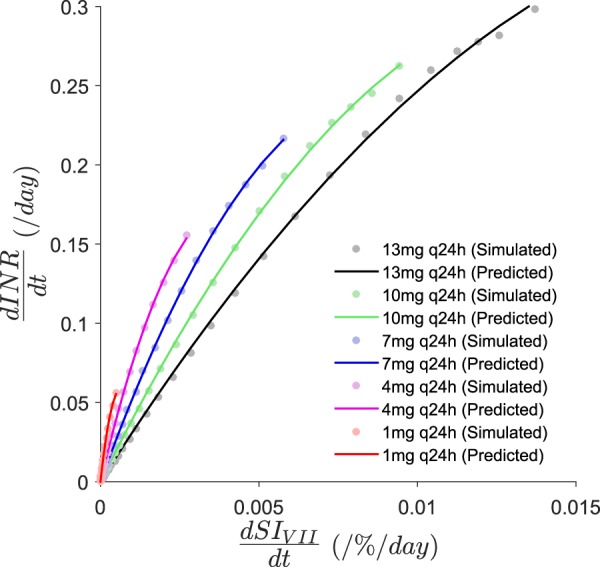
Model fits of the general quadratic function for $\frac{dINR}{dt}$ versus $\frac{dS{I}_{VII}}{dt}$ data. The solid lines are the prediction from the empirical quadratic model (Equation 14) and the filled circles are the data that were simulated from the QSP coagulation network model. Data beyond the model predictions are omitted from the figure. *SI*_*VII*_ is the sensitivity index of INR to factor VII.

Factor VII and INR data were simulated from the coagulation model for different warfarin dosing rates of 1 mg, 4 mg, 7 mg, 10 mg, and, 13 mg daily. $\frac{dINR}{dt}$ and $\frac{dS{I}_{VII}}{dt}$ were computed and a quadratic model was fitted to the $\frac{dINR}{dt}$ versus $\frac{dS{I}_{VII}}{dt}$ data in which nonlinearity was exhibited at high values of $\frac{dS{I}_{VII}}{dt}$ (Fig. 5). Since the y-intercept was approximately zero the following two-parameter model was used:

13 $$\begin{array}{c}l(\frac{dS{I}_{VII}}{dt})=k\times {(\frac{dS{I}_{VII}}{dt})}^{2}+m\times \frac{dS{I}_{VII}}{dt};\,t > 0.\,\end{array}$$

Here, *k* is the second-order coefficient and *m* is the first-order coefficient of the quadratic function. The model provided an adequate fit to the data (Fig. 5). Model fits for alternative models considered are shown in Supplementary Figure S5 in Supplementary Information E. The final parameter estimates for all the different models considered are given in Supplementary Tables S5, S6 and S7 (see Supplementary Information E). Subsequently, the five quadratic functions, each corresponds to a specific warfarin dosing rate, were combined into a single joint function. In this expression, *k* and *m* were empirically expressed as a function of *D*_*ref*_,

14 $$\begin{array}{rcl}l(\frac{dS{I}_{VII}}{dt}) & = & k({D}_{ref})\times {(\frac{dS{I}_{VII}}{dt})}^{2}+m({D}_{ref})\times \frac{dS{I}_{VII}}{dt};\,t > 0\,\\ k({D}_{ref}) & = & -{e}^{{c}_{0}+{c}_{1}\times \mathrm{ln}{D}_{ref}}\\ m({D}_{ref}) & = & {e}^{{s}_{0}+{s}_{1}\times \mathrm{ln}{D}_{ref}},\end{array}$$

where, *c*_*i*_ and *s*_*i*_ are parameters defining the dose-dependency of *k* and *m*. The final parameter estimates are given in Table 2. The fit of the final model for $\frac{dINR}{dt}$ versus $\frac{dS{I}_{VII}}{dt}$ data was evaluated visually by superimposing the model predictions on the simulated data (Fig. 5). Combining Equations 7 and 14 provides:

15 $$\begin{array}{rcl}IN{R}_{SS} & \approx  & IN{R}_{0}+k({D}_{ref})\times {\int }_{0}^{{t}_{SS,INR}}{(\frac{dS{I}_{VII}}{dt})}^{2}dt+m({D}_{ref})\times {\int }_{0}^{{t}_{SS,INR}}\frac{dS{I}_{VII}}{dt}dt;\,t > 0\\ k({D}_{ref}) & = & -{e}^{{c}_{0}+{c}_{1}\times \mathrm{ln}{D}_{ref}}\\ m({D}_{ref}) & = & {e}^{{s}_{0}+{s}_{1}\times \mathrm{ln}{D}_{ref}}.\end{array}$$

**Table 2:** Parameter estimates of the general quadratic function for $\frac{dINR}{dt}$ versus $\frac{dS{I}_{VII}}{dt}$ data.

Function	Final estimate (%RSE)		*r* ^2^
*h*(*D*_*ref*_)	*c*_0_	*c*_1_	0.997
	11.9 (1.03)	−2.05 (3.18)	
*m*(*D*_*ref*_)	*s*_0_	*s*_1_	0.987
	5.26 (1.62)	−0.672 (6.75)	

*D*_*ref*_ is the warfarin daily dose for a typical patient, *k* and *m* are coefficient of the quadratic function, *c*_*i*_ defines the dose-dependency of *k*, and *s*_*i*_ the dose-dependency of *m*, *r*^2^ is the adjusted coefficient of determination, *RSE* the relative standard error, and *SI*_*VII*_ the sensitivity index of INR to factor VII.

Here, the definite integrals can be solved analytically. An algebraic solution to the definite integrals and the *INR*_*SS*_ is given in Supplementary Information F. This relationship however does not hold for the first two days of warfarin initiation since factor VII level is not at equilibrium at this time.

### Prediction algorithm

**Figure 6:**
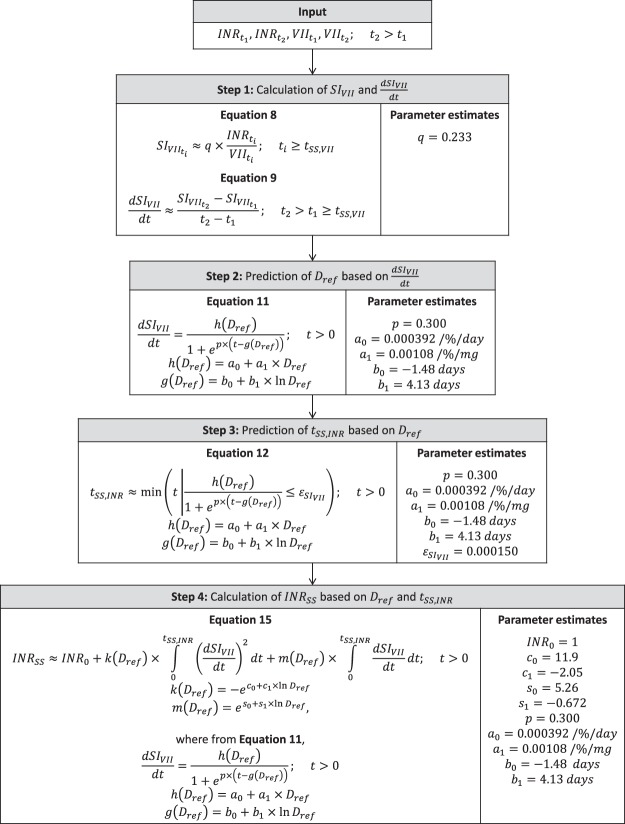
Prediction algorithm of *t*_*SS*,*INR*_, *INR*_*SS*_, and *D*. *a*_*i*_ defines the dose-dependency of *h*, *b*_*i*_ the dose-dependency of *g*, *c*_*i*_ the dose-dependency of *k*, *D* is the warfarin daily dose, *D*_*ref*_ the typical dose that would yield this value of INR (sensitivity of the patient to warfarin), *g* the horizontal shift of the logistic function modelling the $\frac{dS{I}_{VII}}{dt}$ time course, *h* the upper horizontal asymptote of the logistic function modelling the $\frac{dS{I}_{VII}}{dt}$ time course, *INR*_*SS*_ the steady-state INR, *INR*_0_ the INR at baseline, *s*_*i*_ defines the dose-dependency of *m*, *k* and *m* are the coefficient of the quadratic function modelling $\frac{dINR}{dt}$ versus $\frac{dS{I}_{VII}}{dt}$ data, *p* the shape parameter of the logistic function modelling the $\frac{dS{I}_{VII}}{dt}$ time course, *q* the proportionality constant of the linear model for *SI*_*VII*_ versus $\frac{INR}{VII}$ data, *SI*_*VII*_ the sensitivity index of INR to factor VII, *t* the time after warfarin dose change (with index *i* to denote a particular sampling time), and ${\varepsilon }_{S{I}_{VII}}$ the tolerance below which the $\frac{dS{I}_{VII}}{dt}$ corresponds to the steady-state INR.

The mathematical features and parameter values of the 4-step algorithm to predict *t*_*SS*,*INR*_ and *INR*_*SS*_ are shown in Fig. 6. The corresponding MATLAB code for the implementation of the algorithm is available in Supplementary Information G.

## Application of the Prediction Algorithm

The proposed method was applied to a typical simulated patient and two real patients to illustrate proof-of-principle.

### Predicting *INR*_*SS*_ for a virtual patient

Data relating to a typical patient were simulated from a QSP coagulation model^27^ under different dosing scenarios. Factor VII concentrations and INR data at days 3 and 4 were simulated for typical patients who were commenced on warfarin (*D* = 1 mg, 4 mg, 7 mg, 10 mg, or 13 mg daily). These data were analysed according to the 4-step algorithm (Fig. 6) and the predictions of *t*_*SS*,*INR*_ and *INR*_*SS*_ were compared to the simulated values and reported in terms of prediction bias (predicted value minus the simulated value). The simulation and prediction results are shown in Table 3. As expected, the predicted *D*_*ref*_ is approximately equal to *D* since the simulated patient is a typical patient. At different simulated warfarin dosing rates, the bias was within a clinically reasonable range for both the *t*_*SS*,*INR*_ (±2.0 days) and *INR*_*SS*_ (±0.20). One exception was that at a dose of warfarin of 1 mg/day, *t*_*SS*,*INR*_ was under-predicted (−3.7 days). However, this is unlikely to be important clinically considering such a dosing rate is uncommon in clinical practice and furthermore the corresponding prediction of *INR*_*SS*_ was not evidently biased (−0.02).

**Table 3:** Performance of the proposed algorithm to predict the *t*_*SS*,*INR*_ and *INR*_*SS*_ for a simulated typical patient commenced on warfarin at different dosing rates. *D* denotes warfarin daily dose given, *D*_*ref*_ the warfarin daily dose for a typical patient, *INR*_*SS*_ the steady-state INR, and *t*_*SS*,*INR*_ the time to reach steady-state INR.

*D* (mg/day)	Predicted *D*_*ref*_ (mg/day)	*t*_*SS*,*INR*_ (days)			*INR*_*SS*_		
		Simulated^a^	Predicted	Bias	Simulated^a^	Predicted	Bias
1	1.35	11.5	7.8	−3.7	1.41	1.39	−0.02
4	3.97	17.5	15.6	−1.9	2.40	2.28	−0.12
7	6.78	20.5	19.5	−1.0	3.23	3.09	−0.14
10	9.90	22.5	22.3	−0.2	3.98	3.88	−0.10
13	13.3	24.5	24.5	0.0	4.70	4.66	−0.04

^a^Simulated from the QSP coagulation network model^27^.

### Predicting *INR*_*SS*_ for patients

The method was retrospectively assessed in two patients newly commenced on warfarin *D* = 5 mg daily chosen from a previously published dataset^29,30^. This two patients represent all of the patients who have available data that fulfil the requirements to apply the INR prediction method. *t*_*SS*,*INR*_ and *INR*_*SS*_ were predicted using factor VII and INR available at day 3 and day 4. The factor VII and INR profiles of these two patients are shown in Supplementary Information H and the MATLAB scripts with the appropriate input information are provided in Supplementary Information I. The predicted *INR*_*SS*_ was later compared to the observed INR at day 28 (ID A) and day 14 (ID B). Observed INR at later time points were not used as the observations were confounded by non-steady-state conditions introduced by warfarin dosage adjustment. Observed *t*_*SS*,*INR*_ was also unavailable due to sparse sampling of INR. Results of the assessment using real patient data are summarised in Table 4. Both patients showed reasonable agreement in the predicted and observed INRs.

**Table 4:** Performance of the proposed algorithm in predicting the *INR*_*SS*_ for two real patients commenced on warfarin 5 mg q24 h.

ID	*D* (mg/day)	Predicted *D*_*ref*_ (mg/day)	Predicted *t*_*SS*,*INR*_ (days)	Predicted *INR*_*SS*_	Observed INR	Difference in INR
A	5	6.10	18.7	2.9	3.3 (day 28)	−0.4
B	5	7.32	20.0	3.2	3.2 (day 14)	0.0

*D* is the warfarin daily dose given, *D*_*ref*_ the warfarin daily dose for a typical patient, *ID* the patient identifier, and *INR*_*SS*_ the steady-state INR.

## Discussion

We have proposed a factor VII-based approach to predict *t*_*SS*,*INR*_ and *INR*_*SS*_ in patients receiving warfarin. By considering factor VII as the main driver for the INR, this allows the *t*_*SS*,*INR*_ to be determined and from this the *INR*_*SS*_ can be calculated. The proposed method represents a unique approach to predicting the anticoagulant response to warfarin. It incorporates information from factor VII for the prediction of INR and evaluates the INR from the perspective of determining *t*_*SS*,*INR*_. From a practical viewpoint, the proposed method requires timed, paired blood samples of INR and factor VII. When evaluated, the method was associated with clinically reasonable bias.

Our method differs from those reported in the published literature. Several warfarin dosing algorithms quantify the likely anticoagulant response for warfarin maintenance dose prediction using patient characteristics known to influence warfarin dose-response such as body size, age, ethnicity, concomitant drugs, cytochrome P-450 2C9 (CYP2C9) genotype, vitamin K epoxide reductase complex subunit 1 (VKORC1) genotype (see Klein *et al*.^3^ for example). These methods provide no guidance for warfarin dose adjustment once anticoagulant response data (e.g. INR) become available. Other warfarin dosing methods, including traditional initiation nomograms, rely solely on the INR, a composite and blunt measure of anticoagulant response, to guide the prediction of future INR and subsequently, warfarin dose adjustment (see Gedge *et al*.^31^ for example). Bayesian forecasting methods use both prior INR and covariates for INR and warfarin dose prediction (see Wright *et al*.^18^ for example). We propose that measuring the clotting factor data in addition to the INR will be more informative and superior to measuring the INR alone because factors II, VII, and X bridge the gap between warfarin exposure and INR response. The clotting factor data therefore provides a direct measure of the anticoagulant response as well as sensitivity of the patient to warfarin therapy. Here, it is worth noting that measures of anticoagulant response, such as INR and factor VII concentration, will capture between-subject differences in warfarin pharmacokinetics and pharmacodynamics, including those related to CYP2C9 and VKORC1 genotypes. Therefore, in theory, once the time course of anticoagulant response for a given warfarin dose is accounted for, it may not be necessary to consider these and other covariates separately. This would, however, require prospective evaluation. To date, only one published study (Pitsiu and colleagues^32^) considered clotting factor activity for the prediction of warfarin maintenance dose regimens. It was shown that factor VII response measurements alone were adequate to determine dosing requirements although it is important to point out that the sample size is small (*n* = 5) and the use of healthy volunteers potentially limit generalisability of the study’s results to all warfarin users.

**Figure 7:**
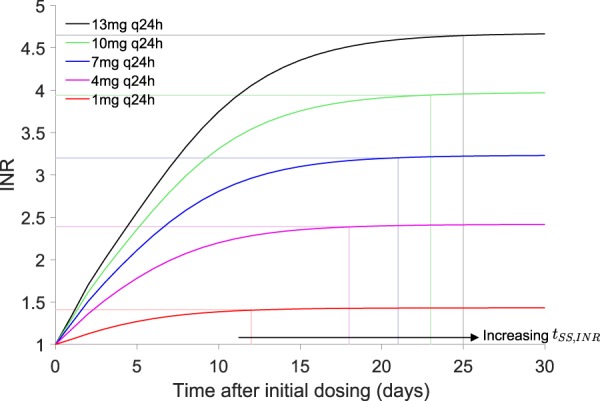
*t*_*SS*,*INR*_ is dose-dependent based on simulations from the QSP coagulation network model^27^. *t*_*SS*,*INR*_ is the time to reach steady-state INR. The vertical lines represent the minimum value of time where the change in INR over time is within a predefined tolerance.

The proposed approach also differs from those previously reported as it attempts to predict *t*_*SS*,*INR*_ and from this, the *INR*_*SS*_ is derived. Based on simulations from the QSP coagulation network model, *t*_*SS*,*INR*_ is observed to be dose-dependent (see Fig. 7). It is thought that the dose-dependency of *t*_*SS*,*INR*_ is due to the inverse relationships between INR sensitivity and clotting factor concentrations i.e. increasing INR sensitivity to clotting factors at low clotting factor concentrations. Since greater warfarin exposure is typically associated with lower clotting factor concentrations, then a disproportionate increase in the INR and delayed achievement of steady-state INR (i.e. longer *t*_*SS*,*INR*_) are expected with high warfarin exposure. In this work, sensitivity of the INR to factor VII was considered and was used to provide an alternative definition for the steady-state INR, which allows the dose-dependency in *t*_*SS*,*INR*_ to be captured and quantified. Predicting *t*_*SS*,*INR*_ allows for a more meaningful interpretation of observed INR response data, which are frequently confounded by non-equilibrium conditions. Then, dosing decisions based on INR measured will be better informed and unnecessary dose adjustments as well as frequent INR monitoring can be avoided.

Our work has several limitations. Due to the absence of explicit functions to describe the relationships between warfarin exposure, *SI*_*VII*_, and INR, the relevant functions were derived empirically to match predictions from a QSP coagulation network model^27^. While the functions were empirical they do reflect the mechanistic processes and hence behave mechanistically. It is important, that the functions described here are not extrapolated beyond the simulations conducted from the QSP model. In this case this would represent doses ranging from 1 to 13 mg/day and INR from 1.4 to 4.7. Despite this limitation the underpinning theory outlined in the Theory Section should remain intact and unaffected. Hence if a scale-reduced version of the QSP model is developed at a later stage then the theory could be applied to a fully mechanistic model. However, it is worth noting that despite the effort to verify the QSP coagulation network model (see Supplementary Information A), it is acknowledged that there remain uncertainties in the applicability of the QSP coagulation network model. It follows that it is possible for a potential inconsistency in the QSP coagulation network model to propagate to the proposed INR prediction method thereby affecting the predictive performance of the method. As a result, it is essential to evaluate and if required, to recalibrate the INR prediction model using prospectively collected warfarin-factor VII-INR data in a large cohort of warfarin patients.

In this study, the observed anticoagulant response was used for individualisation of *D*_*ref*_, which in turn was used for the prediction of *t*_*SS*,*INR*_ and *INR*_*SS*_. *g*(*D*_*ref*_) is not defined when ${D}_{ref}\le 0$. This defines the boundary beyond which the proposed method no longer works and thereby represents a limitation of the method. The limitation, however, is unlikely to be clinically important as the conditions where this would occur, for example if INR became tolerant to factor VII, is unlikely in clinical practice. In addition, the individualisation of *D*_*ref*_ relies on factor VII being at steady state and therefore is confounded by factors that affect the rate of change of factor VII. For instance, an abnormally long degradation half-life of factor VII will lead to a deflated *SI*_*VII*_ estimate and a similarly depressed *D*_*ref*_ estimate, which in turn results in predicted *t*_*SS*,*INR*_ and *INR*_*SS*_ that are biased downward. The proposed method should be adjusted to account for the between patient variability in the degradation half-life of factor VII or alternatively, to sample factor VII at later time points when its steady state has been established. It is important to recognise that the above limitations can also be circumvented by accounting for non-steady state values of factor VII – although this will require additional blood samples to enable quantification of *SI*_*VII*_. Another limitation of the study is that the practical feasibility of the proposed method in routine clinical settings was not considered. For instance, we have assumed that an assay that is appropriately precise and accurate is available to measure blood factor VII concentrations. The proposed method was applied to a typical simulated patient and two real patients to illustrate proof-of-principle. Evaluation of the method in a larger cohort of warfarin patients is not currently possible using retrospectively available data because factor VII is not routinely measured. These results are not generalisable to clinical use. Further work to evaluate the clinical utility of the method using prospectively collected warfarin, factor VII, and INR data would be required. This work would evaluate the predictive performance of the method with respect to both *t*_*SS*,*INR*_ and *INR*_*SS*_.

Finally, a note on sampling times. The sampling times after the achievement of factor VII’s steady-state (e.g. *t*_1_ = *day* 3 and *t*_2_ = *day* 4) are likely to be preferable considering that steady-state factor VII is informative of *INR*_*SS*_^29,33–35^ and that by avoiding sampling on day 1 and day 2, the lag in the onset of factor VII reduction observed in some patients can be bypassed^24,30,32,36^. In addition, *t*_1_ and *t*_2_ should be in temporal proximity to allow accurate approximation of the derivative required for the quantification of *SI*_*VII*_. At this stage, the sampling times are proposed based on heuristic reasons and an optimal design analysis may be able to offer better alternative sampling times – one that minimise the bias in the prediction of *INR*_*SS*_.

It is not our intention to propose a new framework that is instantly applicable for clinical use, but to introduce a new perspective on INR prediction and subsequently, for warfarin dosing. An important future step from this work would be to extend the current method to predict warfarin maintenance dose. This can be achieved by (1) mathematical rearrangement and adaptation of the current method to solve for dose and (2) setting up a dose individualisation algorithm (perhaps Bayesian) as a dose prediction method that incorporates a bivariate response variable. In addition, it is likely that the algorithm can be simplified further, for instance, the number of samples and sampling times for blood samples of factor VII and INR that give the most accurate prediction of *t*_*SS*,*INR*_ and *INR*_*SS*_ could be determined.

## Conclusions

A conceptually different approach for the prediction of future INR has been proposed. The method was associated with minimal bias and its use was illustrated using patient data supporting a proof-of-principle. The proposed method represents a unique approach to predict the INR. It considers factor VII as the main driver for INR and furthermore, it represents the first work to evaluate the INR from the *t*_*SS*,*INR*_ perspective. The prediction of *t*_*SS*,*INR*_ is important as it allows a more meaningful interpretation of the observed INR response – i.e. one that is not confounded by the steady-state status of the INR. Future research to extend the method for warfarin maintenance dose prediction and to assess the predictive performance in a cohort of warfarin patients is required.

## Electronic supplementary material

Supplementary Information

## Data Availability

All data used for model development were simulated from a previously published coagulation network model. The model can be accessed at: http://www.otago.ac.nz/pharmacometrics/downloads/index.html. The data used to demonstrate application of the prediction algorithm was available from a previously published study by McCollum *et al.* 2004. The authors of this work are in the process of seeking permission and obtaining ethical approval to make the aforementioned data publicly available.
