# Supplementary material for: A factor VII-based method for the prediction of anticoagulant response to warfarin
Source: Sci Rep. 2018 Aug 13;8:12041. doi: 10.1038/s41598-018-30516-4 (PMC6089929; doi:10.1038/s41598-018-30516-4)
Supplement: Supplementary file 1 — Supplementary Information [file 41598_2018_30516_MOESM1_ESM.pdf]

Supplementary Information

A factor VII-based method for the prediction of anticoagulant response to warfarin

Qing-Xi Ooi[a*], Daniel F.B. Wright[a], Geoffrey K Isbister[b], Stephen B. Duffull[a]
[a]School of Pharmacy, University of Otago, Dunedin, New Zealand
[b]School of Medicine and Public Health, University of Newcastle, Newcastle, New South Wales, Australia

***Corresponding author**
Qing Xi Ooi
ooiqi725@student.otago.ac.nz

Supplementary Information A Evaluation of assumptions

Rationale and aim:
Here we evaluate the assumptions involved with the proposed method with respect to the probability and the impact of assumption violation.

Assumptions:
The conceptual basis of SI_{VII} to functionally approximate the INR and subsequently to provide a theoretical definition of the steady-state INR was built on the following key assumptions:

- **Assumption 1**: Simultaneous reduction in factors II, VII, and X leads to less than additive increases in the INR
- **Assumption 2**: The most deficient clotting factor drives the INR
- **Assumption 3**: Under non-steady-state INR conditions, factor VII is always the most deficient
- **Assumption 4**: Non-steady-state INR is the most sensitive to factor VII
- **Assumption 5**: The QSP coagulation network model [1] is adequate in describing the warfarin-clotting factors-INR relationship
- **Assumption 6**: The simulated clotting factors-INR time course is representative of that of typical patients initiated with warfarin

Methods:
Each assumption was evaluated independently. Initially, each assumption was reinterpreted in a way that allowed it to be qualified or quantified. Where possible those assumptions that were deemed to be testable were tested. The probability of assumption violation was tested mainly via simulations from the QSP coagulation network model [1] or from an existing warfarin-clotting factors model [2]. The impact of assumption violation was evaluated in terms of whether a violation in the assumption worsens the approximation of the INR by SI_{VII}. The impact of assumption violation was deduced based on logical reasoning or tested using sensitivity analysis. Then, conditioned on the test results, ratings for the probability and the impact of assumption violation were assigned according to the rating scale shown in **Supplementary Table S1**. Finally, based jointly on the probability and impact of assumption violation, an overall risk rating was assigned for each assumption. The same scale for the overall risk as for impact was used.

Supplementary Table S1 Rating scales for the probability and impact of assumption violation

Probability of assumption violation	Impact of assumption violation
Very unlikely	Insignificant
Unlikely	Minor
Possible	Moderate
Probable	Major
Certain	Critical

Results:
The results of evaluation of assumptions are summarised in **Supplementary Table S2**. For all the assumptions, although given the high impact nature of violation, the Probability of violation is low and hence the risk of assumption violation is considered on the whole to be (at worst) minor. Then, since all the assumptions are found to have either insignificant or minor risk of violation, the use of this method for determining INR_{SS} after warfarin dosing was considered to be justified. Details of the methods and results of the assumption evaluation is provided below in **Supplementary Table S2**.

Supplementary Table S2 Results of risk assessment of assumption violation

Assumption	Probability of assumption violation	Impact of assumption violation	Overall risk of assumption violation
1	Very unlikely	Major	Insignificant
2	Very unlikely	Major	Insignificant
3	Very unlikely	Moderate	Insignificant
4	Very unlikely	Major	Insignificant
5	Unlikely	Critical	Minor
6	Unlikely	Critical	Minor

Assumption 1: Simultaneous reduction in factors II, VII, and X leads to less than additive increases in the INR:

Interpretation

$$\Delta INR_{\downarrow i \cap \downarrow j} < (\Delta INR_{\downarrow i} + \Delta INR_{\downarrow j}); \quad i,j \in \{II, VII, X\}, i \neq j \qquad (S1)$$

Here, $\Delta INR_{\downarrow i \cap \downarrow j}$ is the INR response to a simultaneous reduction in two different clotting factors. $\Delta INR_{\downarrow i}$ and $\Delta INR_{\downarrow j}$ represent the INR response to a reduction in a single clotting factor.

Probability of assumption violation

Testability and methods

Testable. Isobolograms of pairwise combinations of factors II, VII, and X with respect to the INR were constructed via simulation from the QSP coagulation network model [1]. All points on the same isobologram correspond to the same INR (e.g. INR=2.5). Points that fall below the additivity line indicate supra-additivity interactions whereas points that are located above the additivity line correspond to subadditivity interactions.

Rating and results

Assumption violation = very unlikely. The isobologram analysis showed that simultaneous reduction in two clotting factors leads to less than additive increase in the INR (See **Supplementary Figure S1**).

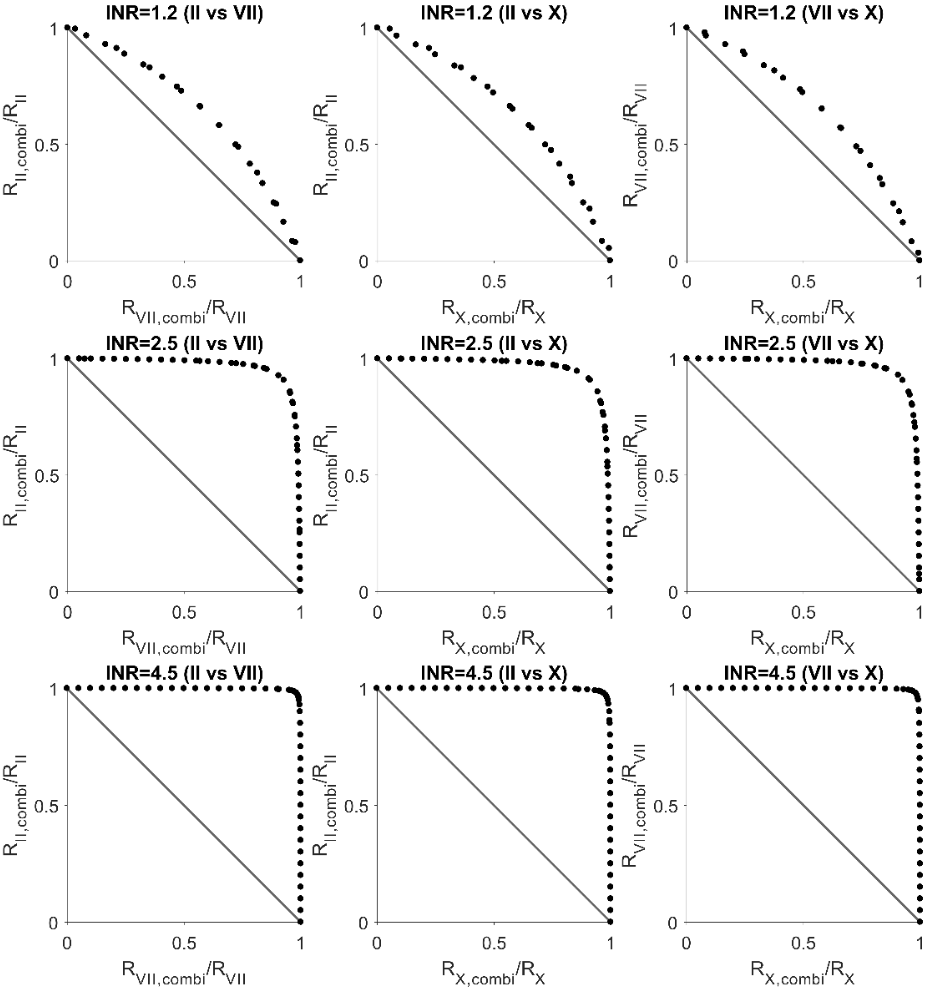

Supplementary Figure S1 Isobolograms of pairwise combination of factors II, VII, and X for different INRs. The black circles correspond to pairwise combination of factors II, VII, or X that gives a predetermined INR. The grey solid line is the additivity line. $R_{F,combi}$ refers to reduction in one clotting factor when another clotting factor is simultaneously reduced, R_F reduction in a single clotting factor.

Impact of assumption violation

Rating and results
Impact = Major. This was rated based on logical reasoning. If the effects of factors II, VII, and X on the INR are additive or supra-additive, contribution of factors II and X to INR will be significant then monitoring of factor VII alone to inform the INR is unlikely to be adequate.

Overall risk assessment
Risk = Insignificant. Even given the high impact nature of violation of this assumption the probability of violation is very low and hence the risk is considered to be negligible.

Assumption 2: The most deficient clotting factor drives the INR:

Interpretation
Of factors II, VII, and X, the most deficient clotting factor is the most important determinant of the INR.

Probability of assumption violation
Testability and methods
Testable. Isobolograms of pairwise combination of factors II, VII, and X with respect to the INR was constructed via simulation from the coagulation network model [1]. All points on the same isobologram correspond to the same level of effects (e.g. INR=2.5).
Rating and results
Assumption violation = Very unlikely. At high INR (e.g. INR=2.5 or INR=4.5), vertical and horizontal segments were observed on the isobologram (see **Supplementary Figure S1**). This showed that if either one of the factors II, VII, and X is sufficiently deficient, then the same INR will occur regardless of the concentration of other clotting factors. Hence, the most deficient clotting factor was considered the principal driving force behind the INR.

Impact of assumption violation
Testability and methods
Not-testable. This is because significant structural change to the coagulation network model, which is unlikely to be consistent with known physiology, is required to make all factors II, VII, and X to drive the INR equally.
Rating and results
Impact = Major. This was rated based on logical reasoning. If factors II, VII, and X all drive the INR considerably, monitoring factor VII alone to inform the INR is unlikely to be adequate.

Overall risk assessment
Risk = Insignificant. Even given the high impact nature of violation of this assumption the probability of violation is very low and hence the risk is considered to be negligible.

Assumption 3: Under non-steady-state INR conditions, factor VII is always the most deficient:

Interpretation

$$1 - P\left(\left(t_{\frac{1}{2},VII} < t_{\frac{1}{2},X}\right) \cap \left(t_{\frac{1}{2},VII} < t_{\frac{1}{2},II}\right)\right) < \alpha; \quad \alpha = 0.05 \qquad \text{(S2)}$$

Here, $t_{\frac{1}{2},F}$ represents the degradation half-life of factors II, VII, or X and α is the significance level for hypothesis testing.

Probability of assumption violation

Testability and methods

Testable. $t_{\frac{1}{2}}$ of factors II, VII, and X for 1000 individuals were simulated using relevant parameters (e.g. the degradation rate constant of factors II, VII, and X and the corresponding variance-covariance matrix of between subject variability in parameters) from a published warfarin-clotting factor model [2]. An overarching hypothesis to test was as follows:

$$H_0: \left(t_{\frac{1}{2},VII} \geq t_{\frac{1}{2},X}\right) \cup \left(t_{\frac{1}{2},VII} \geq t_{\frac{1}{2},II}\right) \qquad (S3)$$
$$H_A: \left(t_{\frac{1}{2},VII} < t_{\frac{1}{2},X}\right) \cap \left(t_{\frac{1}{2},VII} < t_{\frac{1}{2},II}\right).$$

Here, H_0 is the null hypothesis and H_A is the alternative hypothesis. Independent testing of $H_0: t_{\frac{1}{2},VII} \geq t_{\frac{1}{2},X}$ and $H_0: t_{\frac{1}{2},VII} \geq t_{\frac{1}{2},II}$ were required. Each of these hypotheses was tested using a Wilcoxon-signed rank test at $\alpha = 0.025$ (one-tailed and with Bonferroni correction).

Rating and results

Assumption violation = Very unlikely. $\left(t_{\frac{1}{2},VII} - t_{\frac{1}{2},X}\right)$ centred at median -35.0 (IQR -39.9, -30.8) hours. $t_{\frac{1}{2},VII}$ was significantly lower than $t_{\frac{1}{2},X}$ ($Z = -27.4, p < 0.001$). In addition, $\left(t_{\frac{1}{2},VII} - t_{\frac{1}{2},II}\right)$ centred at median -50.7 (IQR -59.0, -43.4) hours. $t_{\frac{1}{2},VII}$ was significantly lower than $t_{1/2,II}$ ($Z = -27.4, p < 0.001$). Taken together, these results provided sufficient evidence to reject the $H_0: \left(t_{\frac{1}{2},VII} \geq t_{\frac{1}{2},X}\right) \cup \left(t_{\frac{1}{2},VII} \geq t_{\frac{1}{2},II}\right)$.

Impact of assumption violation

Testability and methods

Testable. $k_{out,VII}$ of the coagulation network model was reduced so that $t_{\frac{1}{2},VII} > t_{\frac{1}{2},X}$. Then, SI_{II}, SI_{VII}, and SI_X, respectively, were derived from INR and clotting factors data simulated from the coagulation network model [1] using the following equation:

$$SI_F = \left|\frac{\partial INR}{\partial F}\right|; \quad F \in \{II, VII, X\}. \qquad (S4)$$

$SI_{II}/SI_{VII}/SI_X$ versus time were plotted and qualitatively compared to the INR versus time plot (see right-hand column of **Supplementary Figure S2**).

Rating and results

Impact = Moderate. When factor X replaced factor VII as the most deficient factor during the non-steady-state INR, the time course of SI_{VII} no longer bears similarity to that of the INR. Instead, the time course of SI_X appears similar to that of the INR. It appears that what matters is that the SI_F for the clotting factor with the shortest degradation half-life is used as it provides the best approximation to the INR.

Overall risk assessment

Risk = Insignificant. Even given the high impact nature of violation of this assumption the probability of violation is very low and hence the risk is considered to be negligible.

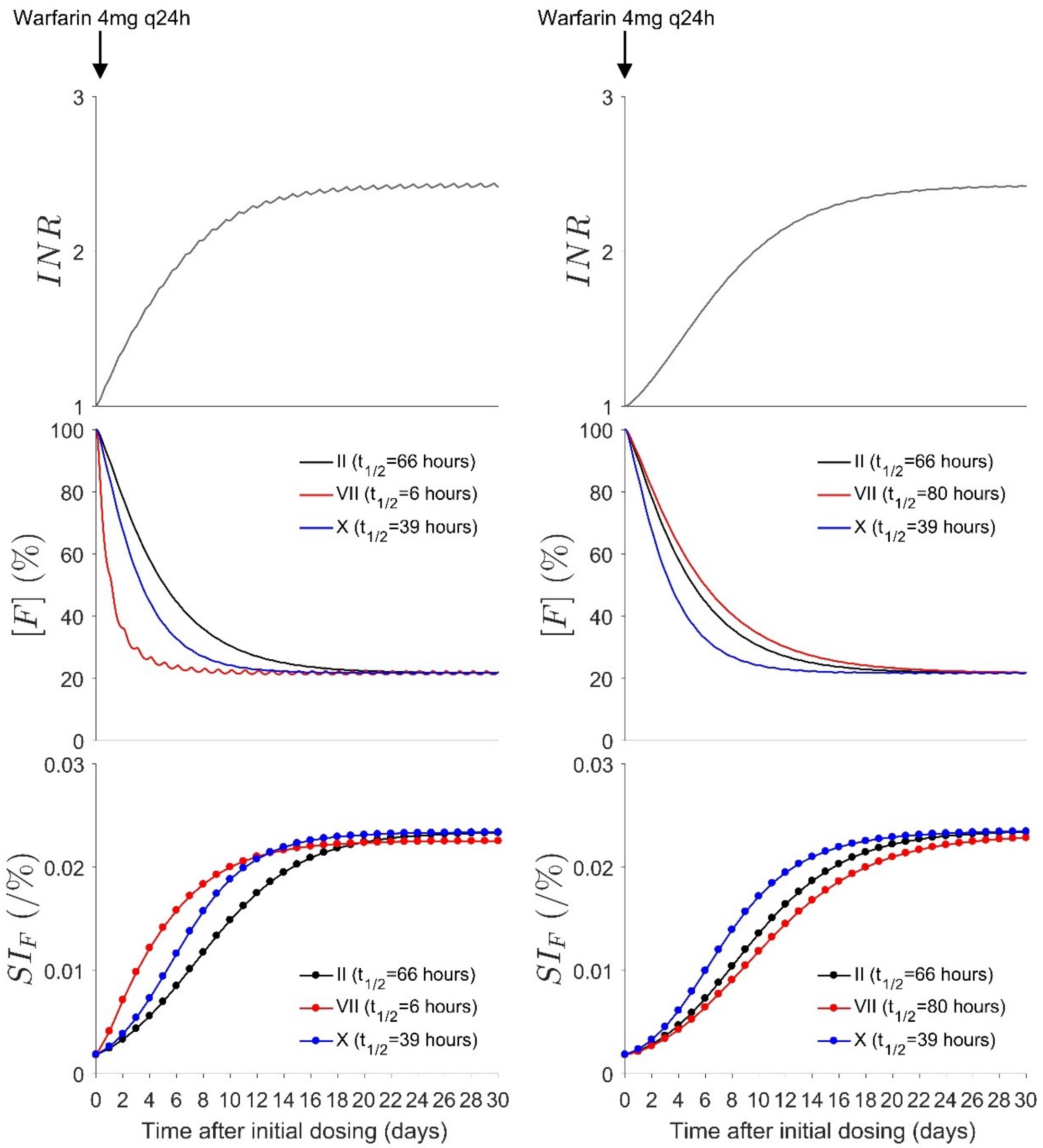

Supplementary Figure S2 The simulated time course of INR, and factors II, VII, and X, and SI_{II}, SI_{VII}, and SI_X following initiation of 4mg warfarin q24h. The two columns of plots differ with respect to the $t_{\frac{1}{2}}$ for factor VII used for simulations. The left-hand column corresponds to $t_{\frac{1}{2},VII} = 6\ hours$ and the right-hand column refers to a hypothetical $t_{\frac{1}{2},VII} = 80\ hours$. SI_F is the sensitivity index of INR to a specific clotting factor and $t_{\frac{1}{2},F}$ the degradation half-life of a particular clotting factor.

Assumption 4: Non-steady-state INR is the most sensitive to factor VII:

Interpretation
SI_{VII} is greater in magnitude compared to both SI_{II} and SI_X at all time points during the non-steady-state INR.

Probability of assumption violation
Testability and methods
Testable. SI_{II}, SI_{VII}, and SI_X, respectively, were derived from INR and clotting factors data simulated from the coagulation network model [1]. $SI_{II}/SI_{VII}/SI_X$ versus time were plotted. At each time point during non-steady-state INR, the magnitude of SI_{VII} was compared to that of SI_X and SI_{II}, respectively (see left-hand column of **Supplementary Figure S2**).
Rating and results
Assumption violation = Very unlikely. It was observed that SI_{VII} is larger in magnitude compared to SI_{II} and SI_X at all time points during the non-steady-state INR.

Impact of assumption violation
Testability and methods
Testable. $k_{out,VII}$ of the coagulation network model was reduced so that $t_{\frac{1}{2},VII} > t_{\frac{1}{2},X}$. Then, SI_{II}, SI_{VII}, and SI_X, respectively, were derived from INR and clotting factors data simulated from the coagulation network model [1]. $SI_{II}/SI_{VII}/SI_X$ versus time were plotted and qualitatively compared to the INR versus time plot (see right-hand column of **Supplementary Figure S2**).
Rating and results
Impact = Major. When factor X replaced factor VII as the most deficient factor during the non-steady-state INR, the time course of SI_{VII} no longer bears similarity to that of the INR. Instead, the time course of SI_X appears similar to that of the INR. It appears that what matters is that the SI_F for the clotting factor with the shortest degradation half-life is used as it provides the best approximation to the INR.

Overall risk assessment
Risk = Insignificant. Even given the high impact nature of violation of this assumption the probability of violation is very low and hence the risk is considered to be negligible.

Assumption 5: The QSP coagulation network model [1] is adequate in describing the warfarin-clotting factors-INR relationship:

Interpretation
The QSP coagulation network model is able to produce physiologically-sound simulated profiles for factors II, VII, X, and INR following warfarin initiation.

Probability of assumption violation
Testability and methods
Testable. Factors II, VII, X, and INR were simulated from the QSP coagulation network model [1]. The simulated data were compared to external data (n=17) [2,3]. Individual observed/simulated data versus time were plotted. It is however, not possible to validate the QSP model and hence it is not possible to fully test this assumption across the whole of the INR generating pathways.
Rating and results
Assumption violation = Unlikely. The simulated data showed reasonably good agreement with the observed data for factors II, VII, X, and INR at the individual level. See **Supplementary Figure S3** for the individual fits of all patients.

Impact of assumption violation
Testability and methods
Testable. No test was carried out as the outcome is self-evident.
Rating and results
Impact = Critical. It is axiomatic that if the QSP model is unable to accurately describe the warfarin-clotting factors-INR relationship, the SI_{VII} derived is unlikely to be fit for the intended purpose.

Overall risk assessment
Risk = Minor. Even given the high impact nature of violation of this assumption the probability of violation is very low and hence the risk is considered to be minor overall.

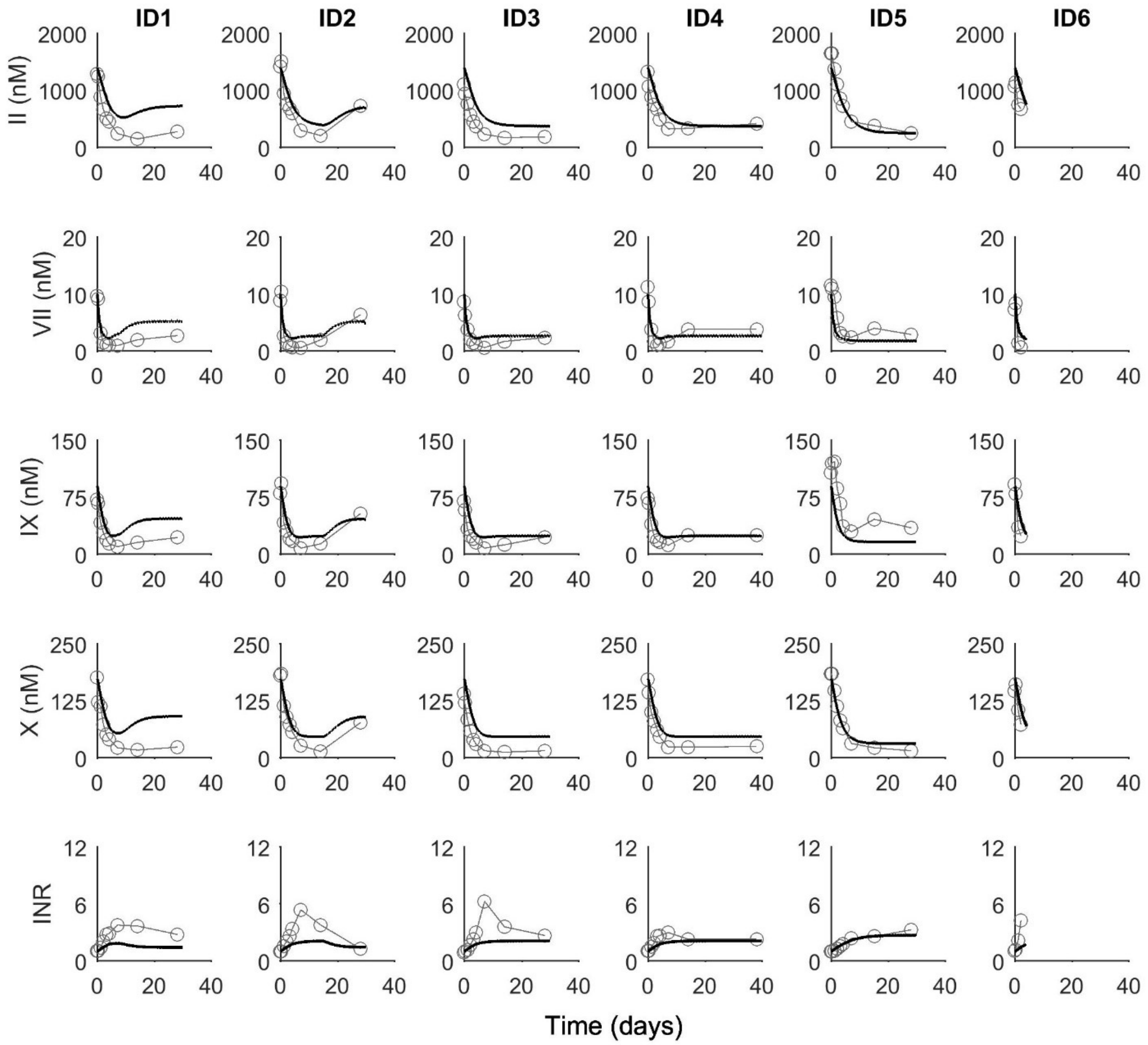

Supplementary Figure S3 Individual fits for the time course of factors II, VII, X, and INR for all 17 patients following warfarin initiation. The black solid lines are prediction by the QSP coagulation network model. The grey open circles are observations.

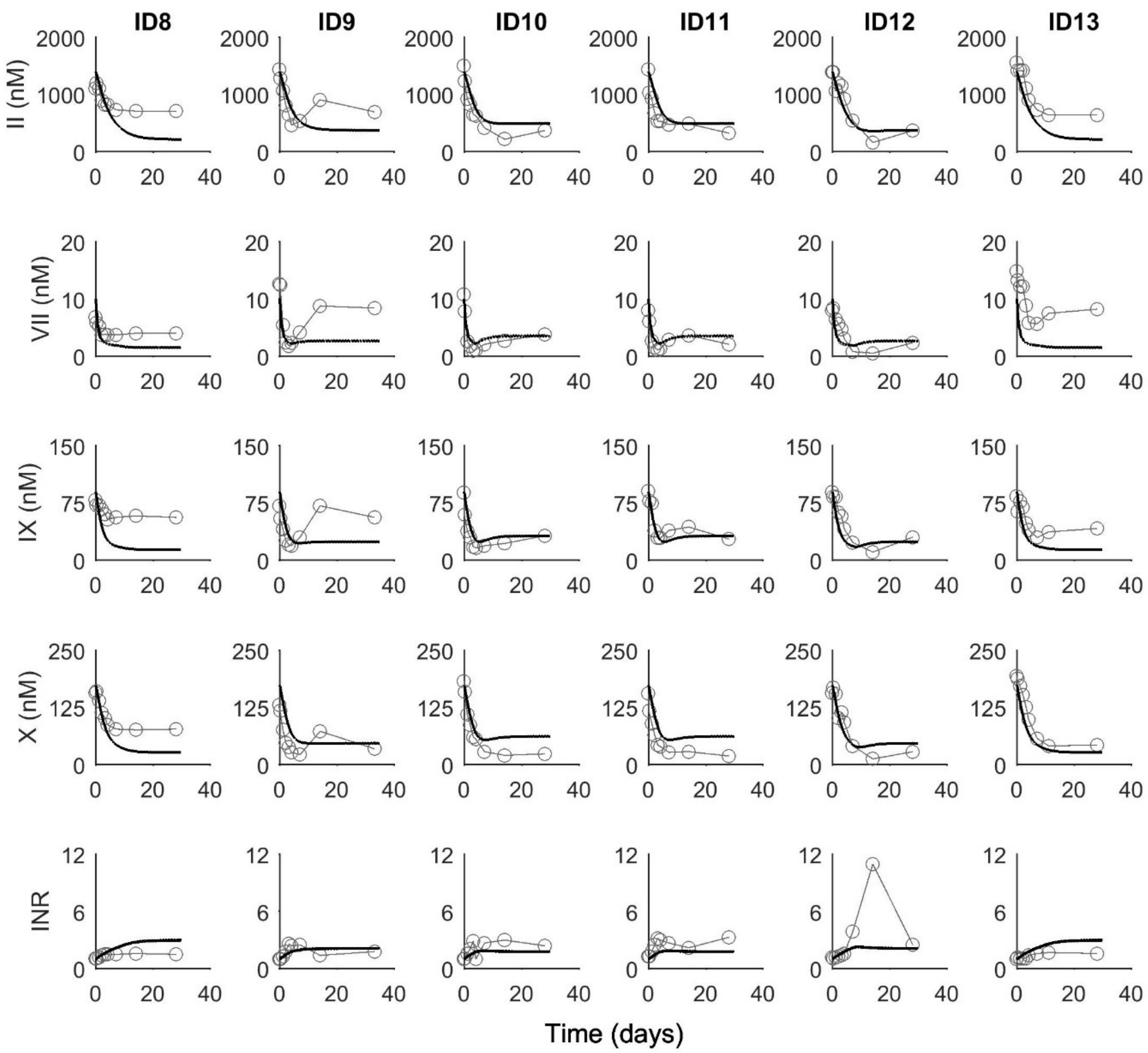

Supplementary Figure S3 Continued.

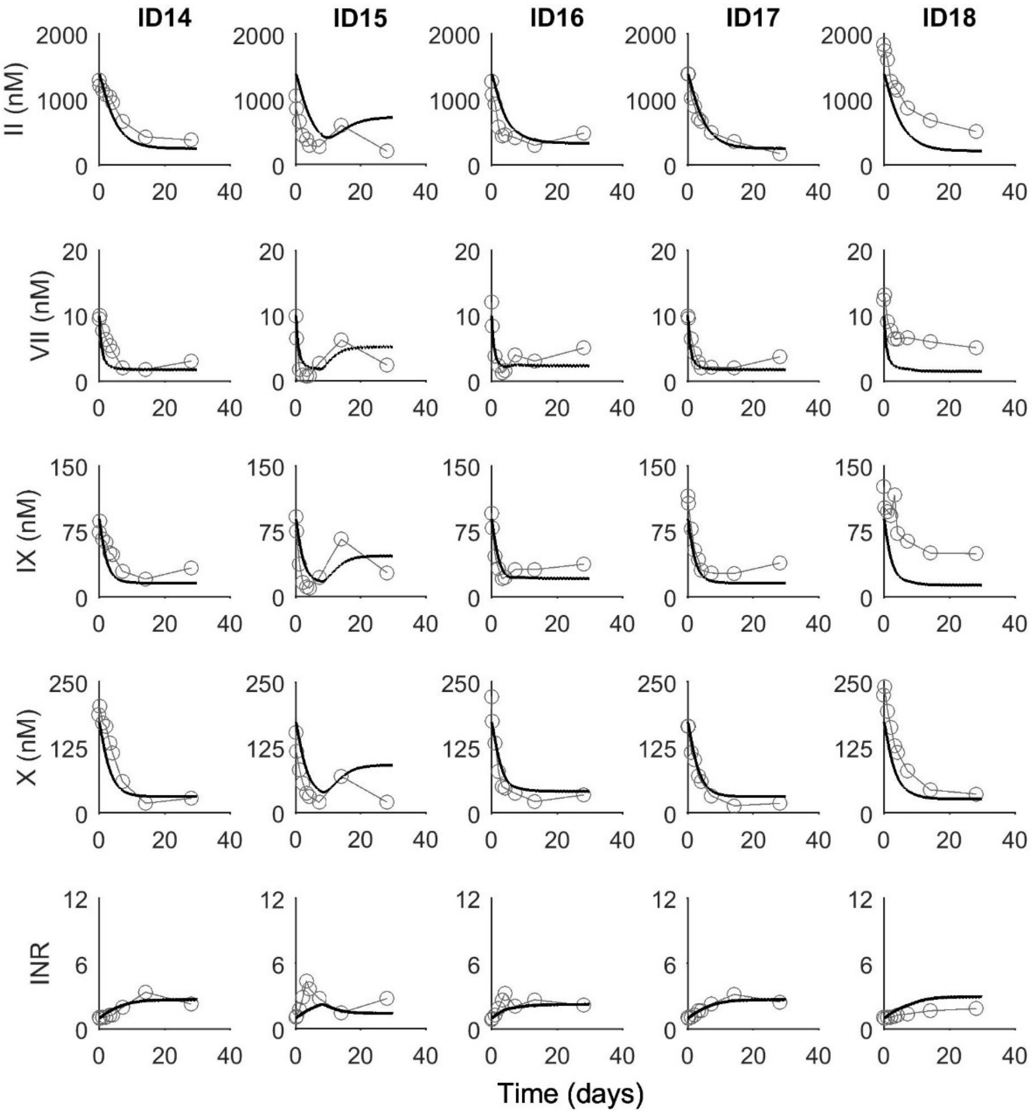

Supplementary Figure S3 Continued.

Assumption 6: The simulated clotting factors-INR time course is representative of that of typical patients initiated with warfarin:

Interpretation
The simulated profiles for factors II, VII, X, and INR are representative of the majority of patients initiated with warfarin.

Probability of assumption violation
Testability and methods
Testable. Factors II, VII, X, and INR were simulated from the QSP coagulation network model [1]. The simulated data were compared to external data ($n = 17$) [2,3]. Individual observed/simulated data versus time were plotted.
Rating and results
Assumption violation = Unlikely. The simulated data showed reasonably good agreement with the observed data for factors II, VII, X, and INR at the individual level. See **Supplementary Figure S3** above for the individual fits of all patients.

Impact of assumption violation
Testability and methods
Testable. No test was carried out as the outcome is self-evident.
Rating and results
Impact = Critical. It is axiomatic that generalisability is limited if the simulation results are unrepresentative.

Overall risk assessment
Risk = Minor. Even given the high impact nature of violation of this assumption the probability of violation is very low and hence the risk is considered to be minor overall.

Supplementary Information B Relationships between warfarin dose, factor VII concentration, INR, and D_{ref}

Supplementary Table S3: Quantification of D_{ref} based on the observed INR response and factor VII concentration for three patients who show different levels of sensitivity to warfarin. In this hypothetical example, all the patients are given the same dose of warfarin. D represents the warfarin dose administered, D_{ref} warfarin dose that would be required by a typical patient to achieve the observed anticoagulant response, *INR* international normalised ratio

Patient	$D\ (mg/day)$	INR	Factor VII concentration	$D_{ref}\ (mg/day)$
Warfarin-sensitive	4	↑	↓	8
Typical	4	↔	↔	4
Warfarin-insensitive	4	↓	↑	2

Supplementary Information C Modelling the time course of $\frac{dSI_{VII}}{dt}$

Supplementary Table S4 Parameter estimates and goodness-of-fit of the logistic model for the time course of $\frac{dSI_{VII}}{dt}$. Both h and g are dose-dependent and p is considered independent. At different warfarin dosing rates, the estimates for p obtained were largely similar i.e. ranging from 0.260 to 0.301 and fixing of p to 0.300 resulted in an almost identical model fit. D_{ref} is the warfarin daily dose for a typical patient, h the upper horizontal asymptote, g the magnitude of horizontal shift, p the shape parameter, r^2 the adjusted coefficient of determination, RSE the relative standard error, and SI_{VII} the sensitivity index of INR to factor VII

D_{ref} (mg/day)	Final estimate (%RSE)			r^2
	h (/%/day)	g (days)	p	
1	0.00200 (6.44)	-1.30 (21.3)	0.300 fixed	0.999
4	0.00442 (1.48)	3.96 (2.48)	0.300 fixed	0.999
7	0.00744 (1.35)	6.33 (1.76)	0.300 fixed	0.999
10	0.0109 (1.10)	8.03 (1.31)	0.300 fixed	0.998
13	0.0149 (0.883)	9.40 (1.00)	0.300 fixed	0.999

[a] Observations for $t = 0.5$ days were excluded from model-fitting.

Supplementary Information D Determination of $\varepsilon_{SI_{VII}}$

In this study, **Equation 15** that is based on SI_{VII} is our approximation to **Equation 8**, which is an approximation to the theoretical gold standard of steady-state INR given by **Equation 1** (via the relationship in **Equation 7**). Based on **Equation 15**, a pre-requisite to the prediction of $t_{SS,INR}$ is the choice of a suitable value of $\varepsilon_{SI_{VII}}$. The choice of $\varepsilon_{SI_{VII}}$ should correctly classify a given INR as non-steady-state (or steady-state) when the INR is truly non-steady-state (or steady-state). The choice of $\varepsilon_{SI_{VII}}$ was based on optimising the receiver operating characteristic (ROC). The ROC curve analysis was conducted in Stata® Version 11.2 (StataCorp LLC, College Station, Texas, USA). **Equation 2**, which represents a practical solution to **Equation 1**, was used as the gold standard to determine the achievement of steady-state INR. Here, a value of ε_{INR} was chosen to represent the target gold standard, in this case $\varepsilon_{INR} = 0.01$, for defining the steady-state INR.

The resulting ROC curve for $\frac{dSI_{VII}}{dt}$ is shown in **Supplementary Figure S4**. From the ROC analysis a choice of $\varepsilon_{SI_{VII}} = 0.000150$ as the tolerance for $\frac{dSI_{VII}}{dt}$ (**Equation 8** and **Equation 15**) corresponds to the steady-state INR. The associated sensitivity and specificity were 100.0% (56/56) and 93.6% (88/94), respectively.

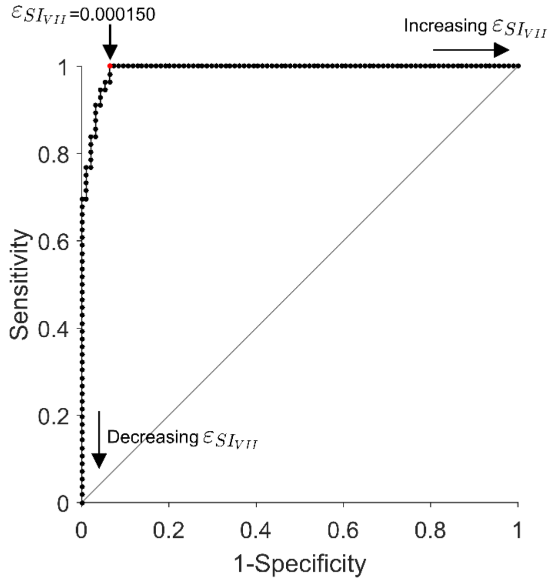

Supplementary Figure S4 ROC curve for choices of the variable $\varepsilon_{SI_{VII}}$ when used to define tolerance in $\frac{dSI_{VII}}{dt}$ for defining the steady-state INR status based on $\frac{dINR}{dt} \leq \varepsilon_{INR}$ (where $\varepsilon_{INR} = 0.01$). The grey solid line represents the reference line for chance performance. SI_{VII} is the sensitivity index of INR to factor VII.

Supplementary Information E Modelling $\frac{dINR}{dt}$ as a function of $\frac{dSI_{VII}}{dt}$

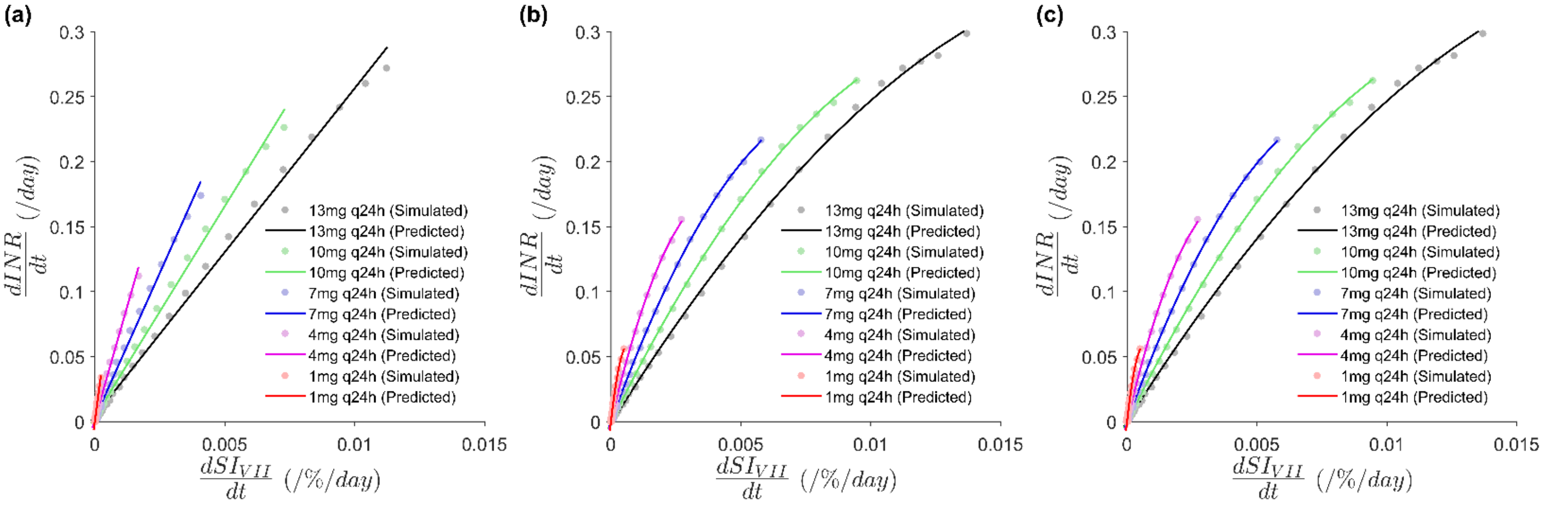

Supplementary Figure S5 Model fits of (a) a 2-parameter linear model, (b) a 3-parameter quadratic model and (c) a 2-parameter quadratic model for the $\frac{dINR}{dt}$ versus $\frac{dSI_{VII}}{dt}$ data. The solid lines are the model predictions and the filled circles are the data that were simulated from the QSP coagulation network model. Data beyond the model predictions are omitted from the figure. SI_{VII} is the sensitivity index of INR to factor VII.

Supplementary Table S5 Parameter estimates and goodness-of-fit of the 2-parameter linear model for the $\frac{dINR}{dt}$ versus $\frac{dSI_{VII}}{dt}$ data for a typical patient. D_{ref} is the warfarin daily dose for a typical patient, k the slope, m the y-intercept, r^2 the adjusted coefficient of determination, RSE the relative standard error, and SI_{VII} the sensitivity index of INR to factor VII.

D_{ref} (mg/day)	Final estimate (%RSE)		r^2
	k (%)	m (/day)	
1	153 (2.65)	0.000770 (36.9)	0.984
4	69.8 (1.55)	0.00195 (31.9)	0.994
7	44.7 (1.45)	0.00290 (33.8)	0.995
10	32.4 (1.36)	0.00376 (34.0)	0.996
13	25.2 (1.28)	0.00446 (34.4)	0.996

[a] Observations for $t = 0.5$, $t = 1.5$, $t = 2.5$, $t = 3.5$ and $t = 4.5$ days were excluded from model-fitting.

Supplementary Table S6 Parameter estimates and goodness-of-fit of the 3-parameter quadratic model for the $\frac{dINR}{dt}$ versus $\frac{dSI_{VII}}{dt}$ data for a typical patient. D_{ref} is the warfarin daily dose for a typical patient, k, m, and w are coefficient of the quadratic model, r^2 the adjusted coefficient of determination, RSE the relative standard error, and SI_{VII} the sensitivity index of INR to factor VII.

D_{ref} (mg/day)	Final estimate (%RSE)			r^2
	k (%^{2}×day)	m (%)	w (/day)	
1	-131000 (0.000000464)	176 (0.856)	0.000527 (41.9)	0.996
4	-9990 (3.17)	83.6 (0.890)	0.000563 (41.7)	1.00
7	-3070 (3.20)	55.1 (0.902)	0.0000830 (435)	1.00
10	-1370 (2.88)	40.9 (0.816)	-0.000695 (60.0)	1.00
13	-735 (4.89)	32.2 (1.39)	-0.00159 (53.2)	1.00

[a] Observations for $t = 0.5$ and $t = 1.5$ days were excluded from model-fitting.

Supplementary Table S7 Parameter estimates and goodness-of-fit of the 2-parameter quadratic model for the $\frac{dINR}{dt}$ versus $\frac{dSI_{VII}}{dt}$ data for a typical patient. D_{ref} is the warfarin daily dose for a typical patient, k and m are coefficient of the quadratic model, r^2 the adjusted coefficient of determination, *RSE* the relative standard error, and SI_{VII} the sensitivity index of INR to factor VII.

D_{ref} (mg/day)	Final estimate (%RSE)		r^2
	k (%^{2}×day)	m (%)	
1	-141000 (8.00)	182 (2.43)	0.996
4	-10300 (2.95)	84.7 (0.767)	1.00
7	-3080 (2.71)	55.2 (0.685)	1.00
10	-1340 (2.59)	40.5 (0.641)	1.00
13	-698 (4.50)	31.6 (1.09)	0.999

[a] Observations for $t = 0.5$ and $t = 1.5$ days were excluded from model-fitting.

Supplementary Information F Symbolic solution for Equation 18

The symbolic solution to the definite integral, $\int_0^{t_{SS,INR}} \left(\frac{dSI_{VII}}{dt}\right)^2 dt$ in **Equation 18** where $\frac{dSI_{VII}}{dt} = \frac{h(D_{ref})}{1+e^{p\times(t-g(D_{ref}))}}$ (**Equation 14**), is given below:

$$\int_0^{t_{SS,INR}} \left(\frac{dSI_{VII}}{dt}\right)^2 dt = \int_0^{t_{SS,INR}} \left(\frac{h(D_{ref})}{1+e^{p\times(t-g(D_{ref}))}}\right)^2 dt \qquad (S5)$$
$$= \left[\frac{h(D_{ref})^2}{p}\left(p \times \left(t - g(D_{ref})\right) - \ln\left|1+e^{p\times(t-g(D_{ref}))}\right| + \frac{1}{1+e^{p\times(t-g(D_{ref}))}}\right)\right]_0^{t_{SS,INR}}.$$

The symbolic solution to the definite integral, $\int_0^{t_{SS,INR}} \frac{dSI_{VII}}{dt} dt$ in **Equation 18** where $\frac{dSI_{VII}}{dt} = \frac{h(D_{ref})}{1+e^{p\times(t-g(D_{ref}))}}$ (**Equation 14**), is given below:

$$\int_0^{t_{SS,INR}} \frac{dSI_{VII}}{dt} dt = \int_0^{t_{SS,INR}} \frac{h(D_{ref})}{1+e^{p\times(t-g(D_{ref}))}} dt = \left[\frac{h(D_{ref})}{p} \times \ln\left|1 - \frac{1}{1+e^{p\times(t-g(D_{ref}))}}\right|\right]_0^{t_{SS,INR}}. \qquad (S6)$$

Then, the full expression for INR_{SS} is given by combining **Equation 18**, **Equation 14**, **Equation S5**, and **Equation S6**:

$$INR_{SS} \approx INR_0 \qquad (S7)$$
$$+k(D_{ref}) \times \left[\frac{h(D_{ref})^2}{p}\left(p \times \left(t - g(D_{ref})\right) - \ln\left|1+e^{p\times(t-g(D_{ref}))}\right| + \frac{1}{1+e^{p\times(t-g(D_{ref}))}}\right)\right]_0^{t_{SS,INR}}$$
$$+m(D_{ref}) \times \left[\frac{h(D_{ref})}{p} \times \ln\left|1 - \frac{1}{1+e^{p\times(t-g(D_{ref}))}}\right|\right]_0^{t_{SS,INR}} ; \quad t > 0$$
$$k(D_{ref}) = -e^{c_0+c_1\times\ln D_{ref}}$$
$$m(D_{ref}) = e^{s_0+s_1\times\ln D_{ref}}$$
$$h(D_{ref}) = a_0 + a_1 \times D_{ref}$$
$$g(D_{ref}) = b_0 + b_1 \times \ln D_{ref}.$$

Supplementary Information G MATLAB code for the implementation of the 4-step algorithm for the prediction of $t_{SS,INR}$ and INR_{SS}

Main file to run (Warf_Algorithm_Main.m)

```
% Proposed algorithm for the prediction of t_SS and INR_SS

%%% No user inputs required

clear all
clc

%%% User inputs required

D=7; % Warfarin daily dose. Unit: mg/day
t1=3; % First sampling time. Unit: days
t2=4; % Second sampling time. Unit: days
INR_t1=1.72; % First INR sample. Unit: none
INR_t2=1.92; % Second INR sample. Unit: none
VII_t1=19.5; % First factor VII sample. Unit: %
VII_t2=17.2; % Second factor VII sample. Unit: %
INR_0=NaN; % Baseline INR. If not available, type 'NaN'. Unit: none.

%%% No user inputs required

Get_Parameters % Read parameter values
Calc_SI_DevSI % Step 1: Calculation of SI and DevSI
Calc_D_ref % Step 2: Prediction of D_ref based on DevSI
Calc_t_SS % Step 3: Prediction of t_SS based on D_ref
Calc_INR_SS % Step 4: Calculation of INR_SS based on D_ref and t_SS
```

Read parameter values (Get_Parameters.m)

```
% Read parameter values

q=0.23269; % Unit: none
p=0.30000; % Unit: none
a_0=0.00039173; % Unit: /%/day
a_1=0.0010791; % Unit: /%/mg
b_0=-1.4805; % Unit: days
b_1=4.1253; % Unit: days
Eps_SI=0.00015000; % Unit: none
c_0=11.942; % Unit: none
c_1=-2.0526; % Unit: none
s_0=5.2636; % Unit: none
s_1=-0.67191; % Unit: none

if isnan(INR_0)==1
INR_0=1; % Unit: none
end
```

Step 1: Calculation of SI_{VII} and $\frac{dSI_{VII}}{dt}$ (Calc_SI_DevSI.m)

```
% Step 1: Calculation of SI and DevSI

SI_t1=q*INR_t1/VII_t1; % Unit: /%
SI_t2=q*INR_t2/VII_t2; % Unit: /%

DevSI=(SI_t2-SI_t1)/(t2-t1) % Unit: /%/day
t_mid=(t1+t2)/2; % Unit: days
```

Step 2: Prediction of D_{ref} based on $\frac{dSI_{VII}}{dt}$ (Calc_D_ref.m)

```
% Step 2: Prediction of D_ref based on DevSI
% Note: An explicit, symbolic expression for D_ref cannot be obtained. D_ref is solved↙
numerically

syms D_ref

h_syms=a_0+a_1*D_ref; % Unit: /%/day
g_syms=b_0+b_1*log(D_ref); % Unit: days

eqn=h_syms/(1+exp(p*(t_mid-g_syms)))==DevSI;
D_ref=double(solve(eqn,D_ref)) % Unit: mg/day
```

Step 3: Prediction of $t_{SS,INR}$ based on D_{ref} (Calc_t_SS.m)

```
% Step 3: Prediction of t_SS based on D_ref
% Note: Since DevSI is a continuous, monotonically declining function over t, then↙
t_SS is the t when h/(1+exp(p*(t_SS-g)))=Eps_SI

h=a_0+a_1*D_ref; % Unit: /%/day
g=b_0+b_1*log(D_ref); % Unit: days

t_SS=1/p*log(h/Eps_SI-1)+g % Unit: days
```

Step 4: Calculation of INR_{SS} based on D_{ref} and $t_{SS,INR}$ (Calc_INR_SS.m)

```
% Step 4: Calculation of INR_SS based on D_ref and t_SS

k=-exp(c_0+c_1*log(D_ref)); % Unit: %*%*day
m=exp(s_0+s_1*log(D_ref)); % Unit: %

% Definite integral of the squared of DevSI
Integ_DevSI_SQR_2=h^2/p*(p*(t_SS-g)-log(1+exp(p*(t_SS-g)))+1/(1+exp(p*(t_SS-g)))); %↙
Upper limit
Integ_DevSI_SQR_1=h^2/p*(p*(0-g)-log(1+exp(p*(0-g)))+1/(1+exp(p*(0-g)))); % Lower↙
limit
Integ_DevSI_SQR=Integ_DevSI_SQR_2-Integ_DevSI_SQR_1; % Unit: /%/%/day

% Definite integral of DevSI
Integ_DevSI_2=h/p*log(1-(1/(1+exp(p*(t_SS-g))))); % Upper limit
Integ_DevSI_1=h/p*log(1-(1/(1+exp(p*(0-g))))); % Lower limit
Integ_DevSI=Integ_DevSI_2-Integ_DevSI_1; % Unit: /%

INR_SS=INR_0+k*Integ_DevSI_SQR+m*Integ_DevSI % Unit: none
```

Supplementary Information H Patient data [2,3]

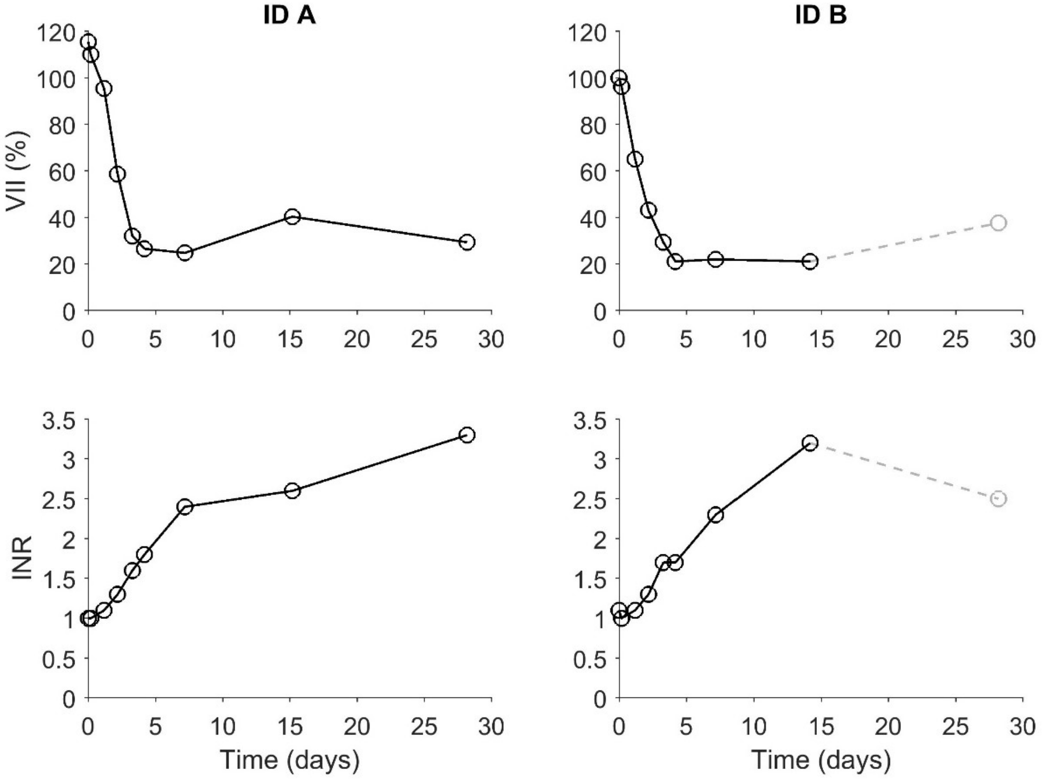

Supplementary Figure S6 Factor VII and INR profiles of the two patients, ID A and ID B, used to illustrate proof-of-concept of the proposed method. Solid black lines relate to warfarin daily dose of 5mg. Dashed grey lines correspond to a likely dose adjustment according to the study protocol [4] after observation of an INR of 3.2 at day 14. The INR observed at day 28 for ID A and day 14 for ID B were compared to that predicted using the proposed method.

Supplementary Information I MATLAB code for the implementation of the 4-step algorithm for the prediction of $t_{SS,INR}$ and INR_{SS} in ID A and ID B

ID A

Main file to run (Warf_Algorithm_Main.m)

```
% Proposed algorithm for the prediction of t_SS and INR_SS

%%% No user inputs required

clear all
clc

%%% User inputs required

D=5; % Warfarin daily dose. Unit: mg/day
t1=3.3; % First sampling time. Unit: days
t2=4.2; % Second sampling time. Unit: days
INR_t1=1.6; % First INR sample. Unit: none
INR_t2=1.8; % Second INR sample. Unit: none
VII_t1=32.1; % First factor VII sample. Unit: %
VII_t2=26.6; % Second factor VII sample. Unit: %
INR_0=1.0; % Baseline INR. If not available, type 'NaN'. Unit: none.

%%% No user inputs required

Get_Parameters % Read parameter values
Calc_SI_DevSI % Step 1: Calculation of SI and DevSI
Calc_D_ref % Step 2: Prediction of D_ref based on DevSI
Calc_t_SS % Step 3: Prediction of t_SS based on D_ref
Calc_INR_SS % Step 4: Calculation of INR_SS based on D_ref and t_SS
```

Read parameter values (Get_Parameters.m)

```
% Read parameter values

q=0.23269; % Unit: none
p=0.30000; % Unit: none
a_0=0.00039173; % Unit: /%/day
a_1=0.0010791; % Unit: /%/mg
b_0=-1.4805; % Unit: days
b_1=4.1253; % Unit: days
Eps_SI=0.00015000; % Unit: none
c_0=11.942; % Unit: none
c_1=-2.0526; % Unit: none
s_0=5.2636; % Unit: none
s_1=-0.67191; % Unit: none

if isnan(INR_0)==1
INR_0=1; % Unit: none
end
```

Step 1: Calculation of SI_{VII} and $\frac{dSI_{VII}}{dt}$ (Calc_SI_DevSI.m)

```
% Step 1: Calculation of SI and DevSI

SI_t1=q*INR_t1/VII_t1; % Unit: /%
SI_t2=q*INR_t2/VII_t2; % Unit: /%

DevSI=(SI_t2-SI_t1)/(t2-t1) % Unit: /%/day
t_mid=(t1+t2)/2; % Unit: days
```

Step 2: Prediction of D_{ref} based on $\frac{dSI_{VII}}{dt}$ (Calc_D_ref.m)

```
% Step 2: Prediction of D_ref based on DevSI
% Note: An explicit, symbolic expression for D_ref cannot be obtained. D_ref is solved↙
numerically

syms D_ref

h_syms=a_0+a_1*D_ref; % Unit: /%/day
g_syms=b_0+b_1*log(D_ref); % Unit: days

eqn=h_syms/(1+exp(p*(t_mid-g_syms)))==DevSI;
D_ref=double(solve(eqn,D_ref)) % Unit: mg/day
```

Step 3: Prediction of $t_{SS,INR}$ based on D_{ref} (Calc_t_SS.m)

```
% Step 3: Prediction of t_SS based on D_ref
% Note: Since DevSI is a continuous, monotonically declining function over t, then↙
t_SS is the t when h/(1+exp(p*(t_SS-g)))=Eps_SI

h=a_0+a_1*D_ref; % Unit: /%/day
g=b_0+b_1*log(D_ref); % Unit: days

t_SS=1/p*log(h/Eps_SI-1)+g % Unit: days
```

Step 4: Calculation of INR_{SS} based on D_{ref} and $t_{SS,INR}$ (Calc_INR_SS.m)

```
% Step 4: Calculation of INR_SS based on D_ref and t_SS

k=-exp(c_0+c_1*log(D_ref)); % Unit: %*%*day
m=exp(s_0+s_1*log(D_ref)); % Unit: %

% Definite integral of the squared of DevSI
Integ_DevSI_SQR_2=h^2/p*(p*(t_SS-g)-log(1+exp(p*(t_SS-g)))+1/(1+exp(p*(t_SS-g)))); %↙
Upper limit
Integ_DevSI_SQR_1=h^2/p*(p*(0-g)-log(1+exp(p*(0-g)))+1/(1+exp(p*(0-g)))); % Lower↙
limit
Integ_DevSI_SQR=Integ_DevSI_SQR_2-Integ_DevSI_SQR_1; % Unit: /%/%/day

% Definite integral of DevSI
Integ_DevSI_2=h/p*log(1-(1/(1+exp(p*(t_SS-g))))); % Upper limit
Integ_DevSI_1=h/p*log(1-(1/(1+exp(p*(0-g))))); % Lower limit
Integ_DevSI=Integ_DevSI_2-Integ_DevSI_1; % Unit: /%

INR_SS=INR_0+k*Integ_DevSI_SQR+m*Integ_DevSI % Unit: none
```

ID B

Main file to run (Warf_Algorithm_Main.m)

```
% Proposed algorithm for the prediction of t_SS and INR_SS

%%% No user inputs required

clear all
clc

%%% User inputs required

D=5; % Warfarin daily dose. Unit: mg/day
t1=3.3; % First sampling time. Unit: days
t2=4.2; % Second sampling time. Unit: days
INR_t1=1.7; % First INR sample. Unit: none
INR_t2=1.7; % Second INR sample. Unit: none
VII_t1=29.4; % First factor VII sample. Unit: %
VII_t2=21.1; % Second factor VII sample. Unit: %
INR_0=1; % Baseline INR. If not available, type 'NaN'. Unit: none.

%%% No user inputs required

Get_Parameters % Read parameter values
Calc_SI_DevSI % Step 1: Calculation of SI and DevSI
Calc_D_ref % Step 2: Prediction of D_ref based on DevSI
Calc_t_SS % Step 3: Prediction of t_SS based on D_ref
Calc_INR_SS % Step 4: Calculation of INR_SS based on D_ref and t_SS
```

Read parameter values (Get_Parameters.m)

```
% Read parameter values

q=0.23269; % Unit: none
p=0.30000; % Unit: none
a_0=0.00039173; % Unit: /%/day
a_1=0.0010791; % Unit: /%/mg
b_0=-1.4805; % Unit: days
b_1=4.1253; % Unit: days
Eps_SI=0.00015000; % Unit: none
c_0=11.942; % Unit: none
c_1=-2.0526; % Unit: none
s_0=5.2636; % Unit: none
s_1=-0.67191; % Unit: none

if isnan(INR_0)==1
INR_0=1; % Unit: none
end
```

Step 1: Calculation of SI_{VII} and $\frac{dSI_{VII}}{dt}$ (Calc_SI_DevSI.m)

```
% Step 1: Calculation of SI and DevSI

SI_t1=q*INR_t1/VII_t1; % Unit: /%
SI_t2=q*INR_t2/VII_t2; % Unit: /%

DevSI=(SI_t2-SI_t1)/(t2-t1) % Unit: /%/day
t_mid=(t1+t2)/2; % Unit: days
```

Step 2: Prediction of D_{ref} based on $\frac{dSI_{VII}}{dt}$ (Calc_D_ref.m)

```
% Step 2: Prediction of D_ref based on DevSI
% Note: An explicit, symbolic expression for D_ref cannot be obtained. D_ref is solved↙
numerically

syms D_ref

h_syms=a_0+a_1*D_ref; % Unit: /%/day
g_syms=b_0+b_1*log(D_ref); % Unit: days

eqn=h_syms/(1+exp(p*(t_mid-g_syms)))==DevSI;
D_ref=double(solve(eqn,D_ref)) % Unit: mg/day
```

Step 3: Prediction of $t_{SS,INR}$ based on D_{ref} (Calc_t_SS.m)

```
% Step 3: Prediction of t_SS based on D_ref
% Note: Since DevSI is a continuous, monotonically declining function over t, then↙
t_SS is the t when h/(1+exp(p*(t_SS-g)))=Eps_SI

h=a_0+a_1*D_ref; % Unit: /%/day
g=b_0+b_1*log(D_ref); % Unit: days

t_SS=1/p*log(h/Eps_SI-1)+g % Unit: days
```

Step 4: Calculation of INR_{SS} based on D_{ref} and $t_{SS,INR}$ (Calc_INR_SS.m)

```
% Step 4: Calculation of INR_SS based on D_ref and t_SS

k=-exp(c_0+c_1*log(D_ref)); % Unit: %*%*day
m=exp(s_0+s_1*log(D_ref)); % Unit: %

% Definite integral of the squared of DevSI
Integ_DevSI_SQR_2=h^2/p*(p*(t_SS-g)-log(1+exp(p*(t_SS-g)))+1/(1+exp(p*(t_SS-g)))); %↙
Upper limit
Integ_DevSI_SQR_1=h^2/p*(p*(0-g)-log(1+exp(p*(0-g)))+1/(1+exp(p*(0-g)))); % Lower↙
limit
Integ_DevSI_SQR=Integ_DevSI_SQR_2-Integ_DevSI_SQR_1; % Unit: /%/%/day

% Definite integral of DevSI
Integ_DevSI_2=h/p*log(1-(1/(1+exp(p*(t_SS-g))))); % Upper limit
Integ_DevSI_1=h/p*log(1-(1/(1+exp(p*(0-g))))); % Lower limit
Integ_DevSI=Integ_DevSI_2-Integ_DevSI_1; % Unit: /%

INR_SS=INR_0+k*Integ_DevSI_SQR+m*Integ_DevSI % Unit: none
```

References

1 Wajima, T., Isbister, G. K. & Duffull, S. B. A comprehensive model for the humoral coagulation network in humans. *Clin Pharmacol Ther* **86**, 290-298, doi:10.1038/clpt.2009.87 (2009).

2 Ooi, Q. X., Wright, D. F., Tait, R. C., Isbister, G. K. & Duffull, S. B. A Joint Model for Vitamin K-Dependent Clotting Factors and Anticoagulation Proteins. *Clin Pharmacokinet*, doi:10.1007/s40262-017-0541-5 (2017).

3 McCollum, D. *et al.* The effect of initiation of oral anticoagulation on protein Z and coagulation activation. *Br J Haematol* **125**, 1 (2004).

4 Tait, R. C. & Sefcick, A. A warfarin induction regimen for out-patient anticoagulation in patients with atrial fibrillation. *Br J Haematol* **101**, 450-454 (1998).
